# A Bayesian Mixed-Methods Analysis of Basic Psychological Needs Satisfaction through Outdoor Learning and Its Influence on Motivational Behavior in Science Class

**DOI:** 10.3389/fpsyg.2017.02235

**Published:** 2017-12-19

**Authors:** Ulrich Dettweiler, Gabriele Lauterbach, Christoph Becker, Perikles Simon

**Affiliations:** ^1^Facutly of Arts and Education, University of Stavanger, Stavanger, Norway; ^2^Department of Sport and Health Sciences, Technical University of Munich, Munich, Germany; ^3^Faculty of Social Science, Media and Sport, Johannes Gutenberg University, Mainz, Germany

**Keywords:** outdoor learning, motivation, children, competence support, science teaching, mixed methods, Bayes factor

## Abstract

Research has shown that outdoor educational interventions can lead to students' increased self-regulated motivational behavior. In this study, we searched into the satisfaction of basic psychological needs (BPN), i.e., autonomy support, the learners' experience of competence, and relatedness, both within the peer group and with their teachers, through outdoor learning. From 2014 to 2016, *n* = 281 students attended “research weeks” at a Student Science Lab in the Alpine National Park Berchtesgaden (Germany). The program is a curriculum-based one-week residential course, centered on a 2-day research expedition. Both before and after the course, students completed a composite questionnaire addressing BPN-satisfaction and overall motivational behavior in relation to the Self-Determination Index (SDI). At the latter time-point, students also reported on their experiences during the intervention. Questionnaire data was analyzed using a set of Bayesian General Linear Models with random effects. Those quantitative measures have been complemented by and contextualized with a set of qualitative survey methods. The results showed that the basic psychological needs influence the motivational behavior in both contexts equally, however on different scale levels. The basic needs satisfaction in the outdoor context is decisively higher than indoors. Moreover, the increment of competence-experience from the school context to the hands-on outdoor program appears to have the biggest impact to students' increased intrinsic motivation during the intervention. Increased autonomy support, student-teacher relations, and student-student relations have much less or no influence on the overall difference of motivational behavior. Gender does not influence the results. The contextualization partly supports those results and provide further explanation for the students' increased self-regulation in the outdoors. They add some explanatory thrust to the argument that outdoor teaching, be it during a residential week, or during occasional but regular sessions as integral part of the “normal” teaching, fosters intrinsic motivational behavior in science with lower secondary students.

## Introduction and theoretical frame

The importance of learning motivation according to the Self-Determination Theory (SDT) (Deci and Ryan, 2000) in the educational context has been widely discussed (Niemiec and Ryan, 2009). In the concept of SDT, one's learning motivational behavior is defined as a self-regulated action. To differentiate between specific loci of causality as a basis of motivational behavior, Deci and Ryan (2000) defined a continuous scale from intrinsic motivation, through extrinsic motivation, to amotivation. Unlike amotivation and intrinsic motivation, extrinsic motivation is subdivided into four distinct types: external, introjected, identified and integrated regulation. In educational contexts, a student is more likely to become intrinsically motivated to learn if a specific task is inherently interesting and enjoyable, and the associated behavior is based on an internal perceived locus of causality (DeCharms, 1968). Within traditional, classroom-based educational practice, the learning content and experience is not always interesting and enjoyable for every student in every lesson; in these instances, in particular, the four extrinsic subcategories are also important to understand students' learning behavior. The identified and integrated types of regulation are associated with an internal perceived locus of causality. For example, the students find values in the learning activity or synthesize activities with other aspects of the self and they regulate their behavior due to an identification with long-term goals, e.g., school-leaving qualification. The introjected and external regulations are associated with a somewhat external perceived locus of causality. For example, students learn to avoid feeling guilty for not having learned or to avoid bad marks and punishment. A-motivation is described as a state of complete absence of motivational behavior. Students' specific motivational behavior can be identified using the Self-Determination Index (SDI) (Vallerand et al., 1997; Levesque et al., 2004; Müller et al., 2007). The SDI is calculated from four motivational domains: intrinsic motivation (InR), identified motivation (IdR), introjected motivation (IjR), and external motivation (ExR):


(1) $$\begin{array}{l} SDI=\left(2\cdot \;InR+IdR\right)\;-\;\left(IjR+2\cdot ExR\right) \end{array}$$

A-motivation and integrated motivation are described as not relevant to determine ones motivational regulation by the SDI-inventory (Ryan and Connell, 1989; Levesque et al., 2004). Recent empirical research suggests, that the relation between ExR and InR can be regarded as a simplex structure (Ünlü and Dettweiler, 2015). This means that motivation regulation types theoretically closer to one another are more strongly interrelated with each other, indicating that the SDT regulatory styles can be linearly ordered along the underlying self-determination continuum.

Within the SDT framework, the students' learning motivation and the possibility to develop intrinsic motivation, is strongly dependent on the satisfaction of basic psychological needs (BPN) for autonomy, competence and relatedness (Deci and Vansteenkiste, 2004; Brandt, 2005). In a BPN-supportive school environment it is more likely that students internalize and apply their motivation to learning. Referring to the continuous scale of motivation, perceived autonomy, for example, decreases from intrinsic motivation over extrinsic motivation to a-motivation. Autonomy in the educational contexts means that students experience their learning behavior as volitional and free from pressure outside of their selves (Niemiec and Ryan, 2009). Teachers' behavior and instruction play important roles in students' basic psychological need satisfaction; the teacher is actively responsible for the creation of BPN-supportive school environments and (Vansteenkiste et al., 2009) and the provision of structure (Sierens et al., 2009). Teachers themselves also experience more or less BPN-supportive working environments. The more that teachers experience autonomy in their daily work, the more they are likely to become of this essential prerequisite, and the more they might then support their students in perceiving autonomy, competence and relatedness (Brandt, 2005; Niemiec and Ryan, 2009). However, due to factors such as pressure on teacher, classroom situation, or teacher personality, a controlling instruction style is often prevalent (Reeve, 2009). The benefits of autonomy-supportive teaching styles both for students' autonomous motivation for learning (Roth et al., 2007), and for students' enhanced engagement in tasks (Reeve et al., 2004) have been described in experimental studies focusing on traditional indoor classrooms. Furthermore, via empirical study, Gnambs and Hanfstingl (2016) showed that the well-documented decline in adolescents' academic intrinsic motivation (e.g., Gottfried et al., 2001; Corpus et al., 2009) is predicted by differences in satisfaction of BPN. The more that BPN were satisfied, the lesser the observed decline in students' intrinsic motivation from 11 to 16 years of age.

Furthermore, recent research has shown that educational interventions using green environments can lead to students' increased integrated and intrinsic motivational behavior. This has been demonstrated both for short-term residential programs (Wang et al., 2004; Sproule et al., 2013; Dettweiler et al., 2015a), and for compulsory curriculum-based outdoor education programs (Becker et al., 2017). However, aspects of students' underlying learning motivation have not been analyzed in such studies, and the methodological quality in outdoor- or adventure educational studies to date has been only moderate (Scrutton and Beames, 2015; Becker et al., 2017).

The current study of a short-term curriculum-based residential outdoor learning course, examined students' learning motivation in the context of Self-Determination Theory. The guiding research question was if and to which degree the satisfaction of basic psychological needs affected self-regulated learning, measured with the Self-Determination Index (SDI). Focus was on students' autonomy support, their experience of competence, and their experience of relatedness both within the peer group and with their teachers.

## Materials and methods

### Participants, intervention

The study group consisted of a convenience sample of *n* = 281 students (168 female, mean age = 12.48 years, *SD* = 1.76; 113 male, mean age = 12.49 years, *SD* = 1.71) from ten classes and five different schools, with a strong bias in the proportion of girls to boys of 6:4 (BF_10_ = 16.48). All students attended lower secondary schools in Germany. For legal reasons, we could not control for the students' family incomes and other socio-economic prevalence. However, socio-cultural backgrounds were considered to be similar across students; and grades in math and German were normally distributed (cf. Supplementary Table 1), suggesting a normal distribution of overall academic achievement in our study group.

### Design and intervention

Data was collected from students in relation to a week of learning in two distinct educational settings: (i) the regular classroom context, and (ii) a curriculum-based 5–6-day residential outdoor learning course—referred to as a “research weeks” (Dettweiler et al., 2015b).

The research weeks combined social learning, personal development, and ecological knowledge on a regional level in an educational concept in order to achieve sustainable learning effects in “global learning.” During the research weeks, the students are split into groups of three to four and develop their knowledge in plant phenology, meteorology and mirco-climatology, glaciology, and pedology—subjects that are part of the official curriculum. Each micro-group is accompanied by either one of the two accompanying teachers, a pre-service teacher student, a staff-member of the science center, or one of the evaluative researchers/authors. On the first 2 days of the course, each group prepares for a research expedition in the lab, i.e., getting to know their specific indicator plant, learning about weather parameters and local weather history, or the geological conditions in the area. Students then complete a 2-day expedition that includes student-led organization of a research protocol and collection of data along a transection of approximately 1,000 m in altitude. Each student group is assigned one facilitator—an accompanying teacher, a student, or scientific staff from the Student Research Center—in order to empower the students to carry out the complex fieldwork and document their findings. A detailed graphical description of the program can be found in Supplementary Image 1.

The research weeks have been delivered at the Student Research Center at Berchtesgaden, run by the Technical University of Munich, from 2013 to 2016, during the months of May to September. Data from 2013 were used as a pilot study (Dettweiler et al., 2015a) and to test and validate the measures used (Dettweiler and Ünlü, 2015). Data from 2014 to 2016 provide the focus of the current study.

### Data collection and measures

Data collection was administered during the week of learning in each educational setting, with the regular classroom context occurring first. Students completed a composite questionnaire comprising a range of validated and bespoke measures. These included an adapted version of the Basic Psychological Need Satisfaction Scale (BPNS) (Deci and Ryan, 2000). The BPNS consists of four scales, i.e., “autonomy support (A),” “competence support (C),” “student-teacher relatedness (RT),” and “student-student relatedness (RS).” The A-scale consists of 11 items and is divided in three sub-scales, asking for “ascertained respect,” “possibilities of choice” and “comprehended reasons.” The C-scale consists of eight items in two subscales, “perceived support,” and “perceived structure.” Each of the relatedness-scales (RT, RS) consists of four items, asking for the quality of social interactions. An adapted version of the Academic Self-Regulation Questionnaire (SRQ-A) (Müller et al., 2007) was used to assess the overall motivational behavior using the Self-Determination Index (SDI).

The scales used for determining motivational behavior and BPN satisfaction were developed and validated for the cohort of the current study. For the SDI, each motivational domain, external regulation (ExR), introjected regulation (IjR), identified regulation (IdR), and intrinsic regulation (InR) was measured on a 5-point Likert scale in subscales of four to five items, resulting, given equation (1), in a range for the SDI from −12 to 12. The BPN measure also used 5-point Likert scales.

As a reduced version of SRQ-A was used, reliability measures for the current study show slightly lower values than the original scales (Müller et al., 2007). This has been accounted for in a mixed-methods approach, and both validity and reliability of the reduced version have been assured despite relatively lower Cronbach α-levels (Dettweiler and Ünlü, 2015). Table 2 displays the reliability measures for the SDI and BPN scales.

In order to gain insight into the students' ideas, concepts, and emotions associated with the respective teaching environment, the questionnaire included a bespoke word-associative method (WAM) task. For this, students were asked to write down three words that spontaneously came to their minds. In the following, we will refer to this class of words as “primary words.” Then, they had the opportunity to elaborate on primary words, with three additional words, referred to as “secondary words.” Similar approaches have been used recently in research examining consumers' perception of specific products (de Andrade et al., 2016). The questionnaires also included open-answer questions about their general experiences in each educational setting. Finally, a group focus interview was conducted with five students, three girls and two boys, following the research week only, to provide insight into the students' experiences concerning competence and autonomy support as well as for relatedness after the intervention.

### Empirical model and data analyses

In order to contextualize and discuss the findings from the various data collections in a mixed-methods research approach, we need to define the empirical model, i.e., specify the order of the different methods and their associated epistemic claims. Following the nomenclature Johnson and Onwuegbuzie (2004) proposed, we can formalize the model as follows:


(2) $$\begin{array}{l} \text{Quan }+\text{ Qual }+\;\left(\text{qual }\to \text{Quan }+\text{ qual}\right) \end{array}$$

where “Quan” stands for “quantitative,” “Qual” for “qualitative.” The arrow “ → ” stands for sequential as opposed to “+,” concurrent. Capital letters denote high priority or weight, lower case letters lower priority or weight. The first “Quan” in Equation (2) refers to the “quantitative” analyses, reported in section Overall Motivational Behavior and Basic Psychological Needs Satisfaction in the two Teaching Settings and The Relative Importance of Basic Psychological Needs Satisfaction with Respect to Increased Self-Regulated Motivational Behavior. “Qual” represents the focus-analysis in section Deductive Focus-Analysis with Respect to Basic Psychological Needs Satisfaction, and (qual → Quan + qual) stands for the word association method in Inductive Categorical Content Analysis and Hierarchical Cluster Analysis of the Primary Words, with “qualitative” text material being analyzed with “quantitative” methodology and exemplified with “qualitative” text data from the open questions (qual).

Stereotypically, “qualitative” or constructivist methodologies aim at a case-by-case understanding of a given empirical data-set, whereas quantitative methodologies seek to generalize certain observations in a subsumption-logical approach (Oevermann, 1979). Moreover, the “quantitative” and “qualitative” research traditions differ with respect to their epistemic claims, with the “quantitative camp” being more on the “realist” side of an epistemological continuum, and the “qualitative camp” more on the “relativist” side (Saint-Mont, 2011), which makes comparisons between findings in both methodologies difficult (Dettweiler, 2015). We used a Bayesian approach for our statistical analyses, as, with its notion of “subjective probability”, it offers an elegant way out of this dilemma (De Finetti, 1974, 1975; Jeffrey, 2004); within Bayesian probability theory, subjective prior beliefs are confronted with data through posterior probability calculations, which inform the researcher if and to which degree the prior belief(s) are warranted or not.

In contrast to the frequentist statistical frame, Bayesian reasoning endorses a subjectivist view, so that the results from those analyses do not make claims about “the world” independent from the researcher's perspective. Ian Hacking has defined this approach with reference to Hilary Putnam's epistemology (Putnam, 1981, 1988) as “internal realism” (Hacking, 1983) which allows, with its pragmatist epistemology, comparative judgments from both, quantitative and qualitative inferences (Hacking, 2001; Jeffrey, 2004).

To account for the complexity of interactions with the SDI and BPN measures, we fitted Bayesian linear mixed models (BLMMs) using the software package bayesFactor (Morey and Rouder, 2015) in R 3.3.2 (2016-10-31) (R Development Core Team, 2008). Without interaction terms, the general model for our analyses for individual *i* is:


(3) $$\begin{array}{l} Y_{ijkl}=\beta_{0}+b_{i}+\beta_{1}\left(gender,j\right)+\beta_{2}\left(context,k\right)\\ \;+\;\beta_{3:6}\left(basic\;needs\;variables,l\right)+\varepsilon_{ijkl}, \end{array}$$

where β_0_ is the intercept and the *b*_*i*_'s are the random intercepts being independent zero mean normally distributed random variables. The residuals ε_*ijkl*_ are also zero mean normally distributed random variables with covariance matrix dependent on the situation as described below. “Context” is an indicator variable showing whether the observation of the four covariates β_3:6_ for each *i* has been made indoors or outdoors. The dependent variable is SDI. All possible variable- and interaction-combinations were determined and sorted according to their explanatory power according to the Bayes factor. The best model was then compared to the following four models, and the effect of each covariate determined by comparing models containing the respective covariate to the intercept-only model. This approach explains the general relative influence of the satisfaction of the basic psychological needs with respect to the two teaching settings and with respect to gender. In order to determine the relative importance of the BPN-variables on the *change* of the SDI from indoor to outdoor, we fitted BLMMs with the difference values with


(4) $$\begin{array}{l} \text{diffSDI }=\;{\text{SDI}}_{\text{outdoor}}\;-\;{\text{SDI}}_{\text{indoor}} \end{array}$$

as the dependent variable and


(5) $$\begin{array}{l} \text{diffC}={\text{C}}_{\text{outdoor}}\;-\;{\text{C}}_{\text{indoor}} \end{array}$$

(6) $$\begin{array}{l} \text{diffA}={\text{A}}_{\text{outdoor}}\;-\;{\text{A}}_{\text{indoor}} \end{array}$$

(7) $$\begin{array}{l} \text{diffRT}={\text{RT}}_{\text{outdoor}}\;-\;{\text{RT}}_{\text{indoor}} \end{array}$$

(8) $$\begin{array}{l} \text{diffRS}={\text{RS}}_{\text{outdoor}}\;-\;{\text{RS}}_{\text{indoor}} \end{array}$$

is notated as:


(9) $$\begin{array}{l} Y_{ijm}=\beta_{0}+b_{i}+\beta_{1}\left(gender,j\right)\\ \;+\;\beta_{2:5}\left(diff\text{\_}basic\;needs\;variables,m\right)+\varepsilon_{ijm}, \end{array}$$

All possible combinations of variables and interactions were again sorted according to their explanatory power by means of the Bayes factor. The best model was then compared to the following four models, and the effect of each covariate determined as described above.

For an adequate determination of the epistemic value of each finding resulting from the above reported models, much depends on the definition of the priors and how they are “informed.”

It is preferable to shape subjective priors on the basis of qualitative information which in turn affect inferences from quantitative data, as had been proposed for mixed-methods research as early as 1994 (Western and Jackman, 1994) and which has been recently been extended to enabling causal inferences (Humphreys and Jacobs, 2015). However, in the current study, the qualitative text corpus appeared to be very diverse so that for the very specific and narrow hypotheses for the quantitative analyses, no such definite informed priors could be specified. But in order to account for this great a priori uncertainty, a random factor was included in all the analyses.

Thus, all Bayesian analyses have been performed with setting default priors in the bayesFactor package (Morey and Rouder, 2015), where Zellner- and Siow- inspired g-priors are placed on effects, but with a separate g-prior parameter for each covariate. A Jeffreys prior is placed on β_0_ and the error term ε, and independent scaled inverse-χ^2^priors with one degree of freedom are placed on the random intercepts b_*i*_ as well as on the fixed-effect covariates β_1-*p*_ as g_1_,…, g_p._ They thus reflect the prior knowledge about the standardized effects (Jeffreys, 1961; Rouder and Morey, 2012; Ly et al., 2016). The Markov Chain Monte Carlo (MCMC) were set on 10,000 MCMC iterations. Effect sizes and relative importance of the variables were calculated with the software package relaimpo (Groemping, 2006). In the interpretation of Bayes Factor values, we followed Jeffreys (1961) and Lee and Wagenmakers (2013) (cf. Table 1).

**Table 1:** Interpretation of Bayes Factor values.

**BF_10_**		**BF_10_**
**Evidence for H_1_**		**Evidence for H_0_^†^**
1	No evidence	1
1–3	Anecdotal evidence	1/3
3–10	Moderate evidence	1/3–1/10
10–30	Strong evidence	1/10–1/30
30–100	Very strong evidence	1/30–1/100
>100	Extreme evidence	<1/100

† *Evidence for H_0_ can also be expressed using BF_01_ = 1/BF_10_*.

In order to analyze the primary and secondary words, two independent coders (UD, GL) translated the German words into English and categorized the words into groups: “negative,” “neutral,” or “positive” and assigned them categories that emerged from the sub-sets in each context, “indoor” and “outdoor.” The two sub-sets of translated and coded words were then compared, and consensus was reached involving a third coder (CB). The resulting databases were then the basis of further analyses.

In a first step, some descriptive statistical analyses, such as the numbers and intersection of emerged codes in the two different teaching contexts and the frequencies of “negatives,” “neutrals,” and “positives” had been determined in order to get a first impression on the data.

In a second step, we explored deeper into the 15 most frequent primary words used in each context. Hereby, we checked the occurrence of those 15 most repeated words in the three dimensions, “negative,” “neutral,” and “positive” with respect to their relative frequencies. This visualizes the emotional connotation of those concepts. In order to understand the context and the function of those words, we clustered them according to their associated secondaries. Therefore, we calculated the Euclidean distance between each of them according to the set of associated secondary words and then normalized the respective distances by setting the mean to zero in order to account for their relative rather than absolute frequencies, due to different sample sizes indoor/outdoor resulting from missing data. The primaries were then clustered using the agglomerative hierarchical algorithm “agnes” in the software package “cluster” in R (Maechler et al., 2016). In order to account for their respective dimensional representation, the primary words in the cluster plots are presented in % gray scale representing their relative positive connotation with black as 100% and white as 0% positive connotation according to their respective secondaries. Finally, the intersection of words in both contexts, indoor and outdoor, have been cross-tabulated by “dimension” to determine the Pearson residuals for each cell and the independent multinomial Bayes factor for the table. With those calculations and visualizations, we were able to statistically compare the categorical text data with respect to the two teaching contexts.

The open questions as well as the group focus-interview were analyzed using content analysis with focus on anchor-examples (Mayring, 2014).

All findings were then contextualized. Since both quantitative and qualitative analyses use the same pragmatist epistemological frame (Hacking, 1983), it is possible to directly compare and contextualize the findings from all analyses without having to prioritize their epistemic values and we can concentrate on their practical significance.

## Results

### Overall motivational behavior and basic psychological needs satisfaction in the two teaching settings

**Figure 1:**
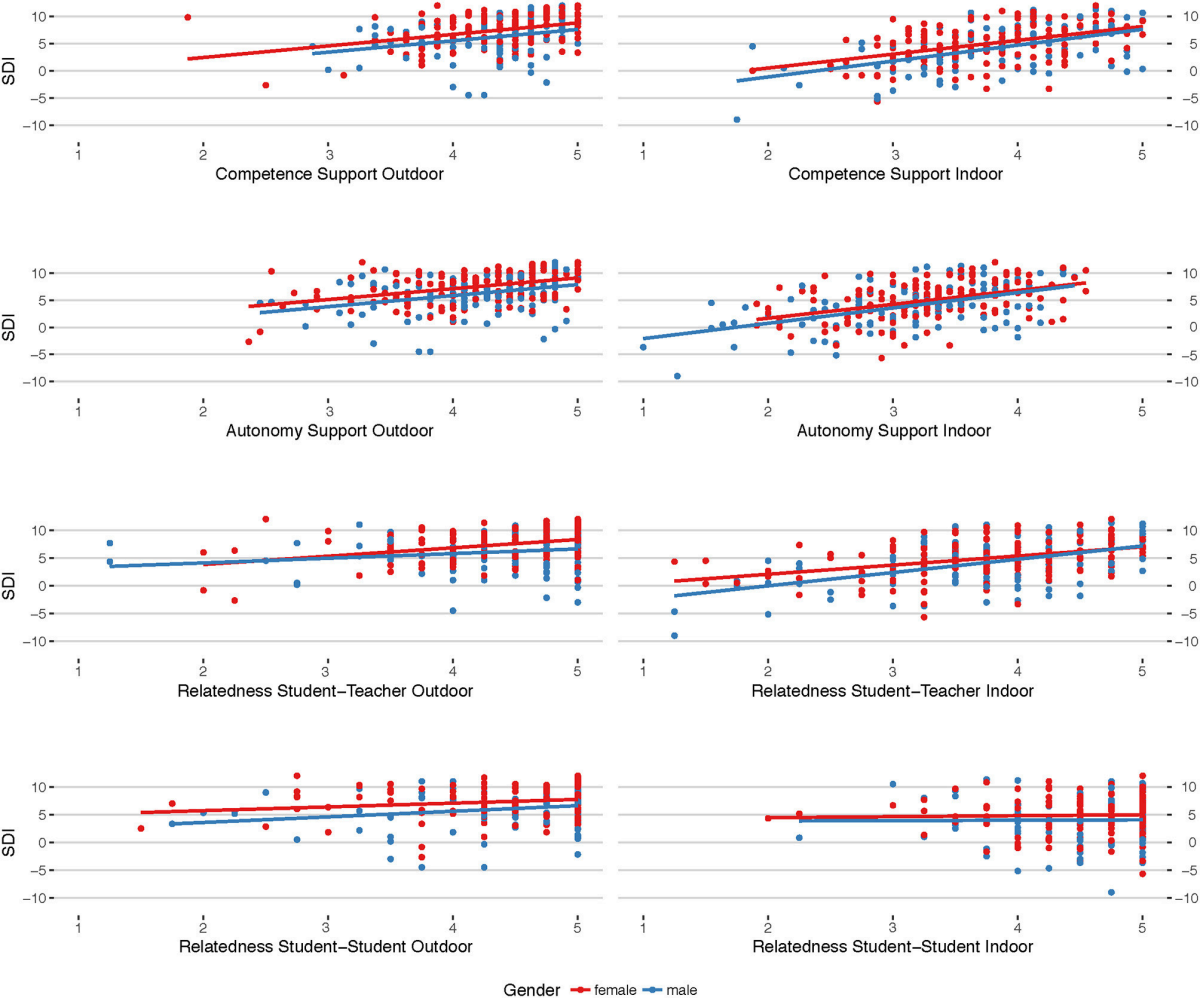
Displayed are the scatterplot matrices for the basic psychological needs variables against the Self-Determination Index (SDI) in both teaching contexts, outdoor (left column) and indoor (right column), with fitted least-square lines. Red dots and lines indicate the girls', blue dots and lines the boys' values. It can be seen that competence- and autonomy- support, as well as student-teacher relatedness correlate most with SDI (slope), irrespective of the context, however, with higher SDI values in the outdoors (intersect).

The results show that the theoretical construct of basic psychological needs satisfaction influences self-regulated learning in both teaching contexts, indoors and outdoors. Figure 1 displays a positive linear relation of BPN-satisfaction and Self-Determination Index (SDI).

The slopes for competence and autonomy support, which are the two most important covariates in the model against SDI, are almost the same in both teaching contexts, with considerable gender effects (intercept), where red dots and lines indicate the girls', blue dots and lines the boys' values. Moreover, it can be seen that the BPN-satisfaction for the outdoor setting is considerably higher than for indoors, resulting in higher SDI's. Bayesian Paired-Samples *T*-tests suggest very strong evidence for the hypotheses, that SDI, competence (C), autonomy (A), and student-teacher relatedness (RT) are higher in the outdoor context (cf. Table 2).

**Table 2:** Descriptive and Inferential Statistical Information on the SDI-Model.

**Model parameters (Reliability measure cronbach's α)**	**Full model (*R*^2^ = 0.307)**	**Bayesian paired-samples** ***t*****-test of covariates factored by gender and context**		**Model with single covariates as fixed effects**	**Descriptives, factored by gender and context**			
	**Bayes factor, if parameter is added to the model (error %)**	**Bayes factor for mean comparison female (error %)**	**Bayes factor for mean comparison male (error %)**	**Effect size *R*^2^ {Relative importance when fitted in the fully factored covariate model}**	**Mean indoor Female (SD)**	**Mean outdoor Female (SD)**	**Mean indoor Male (SD)**	**Mean outdoor Male (SD)**
**DEPENDENT VARIABLE**								
SDI (0.688)	–	2.1 × 10^14^ (< 0.001)	9.4 × 10^3^ (< 0.001)		[*n* = 163] 4.915 (3.410)	[*n* = 162] 7.459 (2.799)	[*n* = 107] 4.034 (4.089)	[*n* = 112] 6.143 (3.452)
**COVARIATES**								
A (0.876)	2.4 × 10^33^ (< 0.001)	2.3 × 10^36^ (< 0.001)	5.6 × 10^18^ (< 0.001)	0.258 {33.01%}	[*n* = 164] 3.266 (0.578)	[*n* = 167] 4.141 (0.584)	[*n* = 107] 3.164 (0.730)	[*n* = 112] 4.144 (0.581)
C (0.797)	3.4 × 10^28^ (< 0.001)	1.9 × 10^20^ (< 0.001)	5.2 × 10^7^ (< 0.001)	0.226 {28,65%}	[*n* = 163] 3.705 (0.717)	[*n* = 167] 4.335 (0.492)	[*n* = 106] 3.796 (0.700)	[*n* = 112] 4.300 (0.472)
RT (0.866)	5.5 × 10^22^ (< 0.001)	5.3 × 10^14^ (< 0.001)	9.7 < 10^9^ (< 0.001)	0.186 {21.03%}	[*n* = 164] 3.738 (0.664)	[*n* = 166] 4.399 (0.664)	[*n* = 107] 3.694 (0.859)	[*n* = 112] 4.424 (0.725)
RS (0.835)	1.11 (< 0.001)	0.131 (< 0.001)	0.108 (< 0.001)	0.009 {0.90%}	[*n* = 164] 4.587 (0.551)	[*n* = 167] 4.531 (0.701)	[*n* = 107] 4.537 (0.561)	[*n* = 112] 4.531 (0.713)
**FACTORS**								
Context	2.68 × 10^11^ (< 0.001)	–	–	0.103 {9.76%}	–	–	–	–
Gender	21.31 (< 0.001)	–	–	0.020 {6.65%}	–	–	–	–

*SD, Standard Deviation; SDI, Self-determination Index; A, Autonomy Support; C, Competence Support; RT, Relatedness Student-Teacher; RS, Relatedness Student-Student. Context has two levels, “indoor” and “outdoor,” as has Gender, with “female” and “male.”*

For student-student relatedness (RS), no evidence can be deemed for differences between the indoor- and outdoor-contexts. The paired-sample analysis for RS is concurrent with the corresponding Bayesian Linear Mixed Model. The best model fit was the model with competence and autonomy support, student-teacher relatedness as co-variates, and gender as factor


(10) $$\begin{array}{ll} Y{\left(SDI\right)}_{ijmno}=\beta_{0}+b_{i}+\beta_{1}\left(gender,j\right)+\beta_{2}\left(A,m\right)+\beta_{3}\left(C,n\right)\\ \; & \;+\;\beta_{4}\left(RT,o\right)+\varepsilon_{ijmno} \end{array}$$

and without interaction terms. RS has virtually no effect; neither has Context, indicating that the mechanisms of the influence of the satisfaction of basic psychological needs is the same in both contexts (cf. Supplementary Figure 1 and Supplementary Table 2).

As the effect-analysis in Table 2 shows, autonomy- (BF_10_ = 2.4 × 10^33^, *R*^2^ = 0.258) and competence- (BF_10_ = 3.4 × 10^28^, *R*^2^ = 0.226) support, as well as student-teacher relatedness (BF_10_ = 5.3 × 10^14^, *R*^2^ = 0.186) were the most important covariates in the model and have an extremely strong effect on the outcome variable SDI. Together, they share about 82% of relative importance in the fully-factored covariate model. The gender effect can be considered small, with girls being higher self-regulated learners in science than boys (BF_10_ = 21.31, *R*^2^ = 0.020), both in the indoor and the outdoor contexts. The corresponding MCMC-iteration plots for the posterior distributions as well as the highest probability density (HPD) intervals can be found in Supplementary Figure 2 and Supplementary Table 3.

### The relative importance of basic psychological needs satisfaction with respect to increased self-regulated motivational behavior

**Figure 2:**
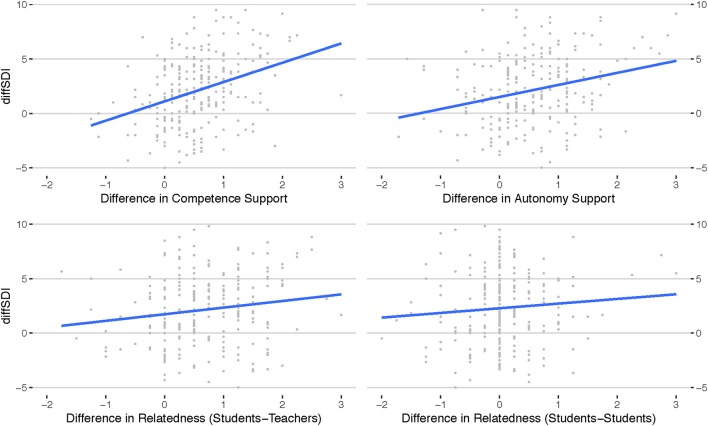
Displayed are the scatterplot matrices for the difference values of the basic psychological needs variables against the difference values of the Self-Determination Index (SDI), with fitted least-square lines. It can be seen that differences in competence support correlates most with difference in SDI (slope effect).

In order to determine the relative importance of each of the basic psychological needs (A, C, RT, and RS) for the change in self-regulated motivational behavior, we can look at the particular difference-BPN-values from the indoor to the outdoor contexts defined in Equations (4–8). The model yields that increase in competence support has by factor 592 the biggest relative importance to explain the variance in increased SDI (BF_10_ = 3.1 × 10^6^, *R*^2^ = 0.144) over the second most important, perceived difference in autonomy support (BF_10_ = 5.2 × 10^3^, *R*^2^ = 0.098). Perceived increase in student-teacher relatedness (BF_10_ = 6.054, *R*^2^ = 0.031) was the third most important component in the model. The importance of students-students relations and gender is not noteworthy (cf. Figure 2 and Table 3). Consequently, model comparison returns that the model


(11) $$\begin{array}{l} Y{\left(diffSDI\right)}_{ij}=\beta_{0}+b_{i}+\beta_{1}\left(diffC,j\right)+\varepsilon_{ij} \end{array}$$

with diffC as the only independent variable fits the data best, being by factor 2.81 more probable than diffC + diffA, yielding about 60% relative importance in the model and explaining about 14% of the variance in increased SDI. The model parameters, their corresponding MCMC-iteration plots for the posterior distributions as well as the highest probability density (HPD) intervals can be found in the Supplementary Material (section Results).

**Table 3:** Descriptive and Inferential Statistical Information on the diffSDI-Model.

**Full Model (*****R***^**2**^ = **0.157)**		**Model with single covariates as fixed effects**	**Descriptives, factored by gender**	
**Model parameters**	**Bayes Factor, if parameter is added to the model (error %)**	**Effect Size *R*^2^ {Relative importance when fitted in the full covariate factored model}**	**Mean Female (SD)**	**Mean Male (SD)**
**DEPENDENT VARIABLE**				
diffSDI	–		[*n* = 158] 2.493 (3.283)	[*n* = 106] 2.096 (4.224)
**COVARIATES**				
diffC	3.1 × 10^6^ (< 0.001)	0.144 {59.22%}	[*n* = 157] 0.590 (0.533)	[*n* = 102] 0.521 (0.766)
diffA	5.2 × 10^3^ (< 0.001)	0.098 {28.18%}	[*n* = 151] 0.519 (0.672)	[*n* = 100] 0.669 (0.947)
diffRT	6.054 (< 0.001)	0.031 {8.61%}	[*n* = 156] 0.675 (0.822)	[*n* = 102] 0.703 (0.923)
diffRS	0.662 (< 0.001)	0.011 {2.66%}	[*n* = 162] −0.0401 (0.719)	[*n* = 104] 0.0192 (0.765)
**FACTOR**				
Gender	0.188 (< 0.001)	0.003 {1.33%}	–	–

*SD, Standard Deviation; diffSDI, Difference Value of Self-determination Index; diffA, Difference Value of Autonomy Support; diffC, Difference Value of Competence Support; diffRT, Difference Value of Relatedness Student-Teacher; diffRS, Difference Value of Relatedness Student-Student. Gender has two levels, “female” and “male.”*

### Findings from the focus group interview as well as the open questions and word associations in the questionnaire

#### Deductive focus-analysis with respect to basic psychological needs satisfaction

##### Competence support

The focus-analysis of the open questions and the group-interview hints at a strong difference in perceived competence support in the two teaching contexts. For the indoor context, we found many reports on lack of understanding due to “bad” teaching. For example, a 12-year-old girl expresses her experience in the following words:

“*When she [the teacher] writes something on the black board, she already starts to explain something new, so that one cannot understand anything. And sometimes, she is speaking so fast…” (15GB18f)*^1^.

This statement is the more remarkable since this girl had grade A in “science” class in her last report before the survey. A 12-year-old girl from another school is very explicit about lack of perceived competence support:

“*With respect to competence support, where it is said that my teachers really know the stuff, I would subtract points. My teacher in mathematics is completely unable to explain stuff” (16SB9f)*.

One should note that her grade in mathematics is B.

Furthermore, one 13-year-old girl, who is also a very good student (grade A in “science”), reports that she often does not understand what the teacher explains. But she tries to understand and learn as much as she can. She continues her statement:

“*I wish that much more would be explained in more detail and clarity so that it does not remain obscure to me. More experiments would help my understanding. But I like this subject” (15SB14f)*.

A 12-year-old boy, with rather mediocre remarks in both, “science” and “mathematics,” shares this demand for experiments, however for other reasons: he thinks that

“*doing stuff, for example with the Bunsen burner, is cool” (15SB1m)*.

Experiments and experiential learning seem to be key factors for perceived competence in the indoors, and being associated with fun. Some of the examples for experiential learning in the “indoor” context are, in fact, referring to learning activities outside the classroom. Those include excursions to a science lab at the university or to an eco-farm, but also short sections of outdoor surveys in the school yard or nearby park. But those are scarce for the “indoor” context and thus highly valued by the students.

The importance of experiments and hands-on-learning can also be corroborated from the reports on the outdoor teaching. Exemplarily, 12-year-old boy describes his best experience in the outdoors with the following words:

“*The hiking was great because the experiments were good, and everything else, too. The only thing that was not so good was that my shoes had been wet the whole time” (15GB30m)*.

The focus group interview shows the same pattern: indoor teaching is perceived difficult as long as the teaching methods are not experiential. One of the interviewed girls reports that she liked it better at the research week because her science teacher at school

*“only copies text from the book, and we hardly learn anything … and then he yells: ‘Zack Zack, now you need to be ready” (14SBxf*^2^*)*.

Her interview partner, also a girl (14SByf), reports that during the outdoor teaching, everyone—teachers, university staff and students, and peers—were relaxed and helpful.

##### Autonomy support

The topic of experiential learning methodology is also relevant for the category “autonomy support.” The students value the fact that they “had less rules” and could “do own research” (15SB22f) or build their “own measurement tool” (16GB19f) during the outdoor teaching. The same holds true for the indoor context: the students most like those things they could autonomously work on, e.g., preparing a presentation on a self-chosen topic (16GB1m) or asking for homework (!) that is self-regulated, e.g., producing a leaf-collection (16GB18m). However, reports on actual perceived autonomy support are less frequent for the indoor context than for the outdoors.

The motive of “freedom” is one that comes up often in the outdoor context. “Learning” is perceived more “free” with the experiential approach during the expedition, and was often associated with the general setting in nature.

“Free” is only mentioned once in the indoor text corpus: one student is reporting experiences from an excursion to a frog-pond, where they were allowed to stroll around “freely” (16SB9f).

In the focus group interview, “freedom,” understood as “autonomy,” is also a dominant concept when it comes to the students' reports on learning. As one interviewed boy recalls:

“*I liked it to have had permission to do everything myself. It's really nice, that one is allowed to think a little and do experiments” (14SBzm)*.

This boy also enjoyed to build his own measurement tool because he likes to do things with his hands.

##### Relatedness

Analysis of the text corpus for “relatedness” reveals only limited information for the indoor context. “Friends” is only notated once in the responses on indoor science class, and this 11-year-old girl tells us in fact something about an outdoor-teaching unit:

“*Our science class teacher [name omitted] changes the subjects a little bit each lesson. This is, on the one hand, very informative, but on the other hand also a little bit confusing. In summertime, she often takes us outside and we should do some surveys and determine stuff. This is very relaxing and we can talk to our friends and stroll around” (16GB13f)*.

Much text can be found with respect to the outdoor-intervention, often paired with the concepts “co-operation” and “fun.” Learning together with friends in this great environment is the main motive in the category “relatedness” in the outdoor context, both with new friends (15SB4f, 15GB4f), or old friends (16MWB28f). Some lamented that splitting groups had prevented them to be with their friends “all the time” (16MWB26f), but a very strong motive is “companionship”:

“*It was great when we arrived at the glacier and could view over the clouds. This was a feeling of freedom and companionship, because we all mastered the hike together” (15SB7f)*.

For both contexts, reference to negative experiences with peers was scarce, such as bullying or noisy classmates that disturbed the respective respondents' concentration.

The same holds true for the reported student-teacher relations. Those are, in both teaching contexts, predominantly positive with only very rare exceptions. Those, however, are reported in rather more extreme words.

#### Inductive categorical content analysis

**Figure 3:**
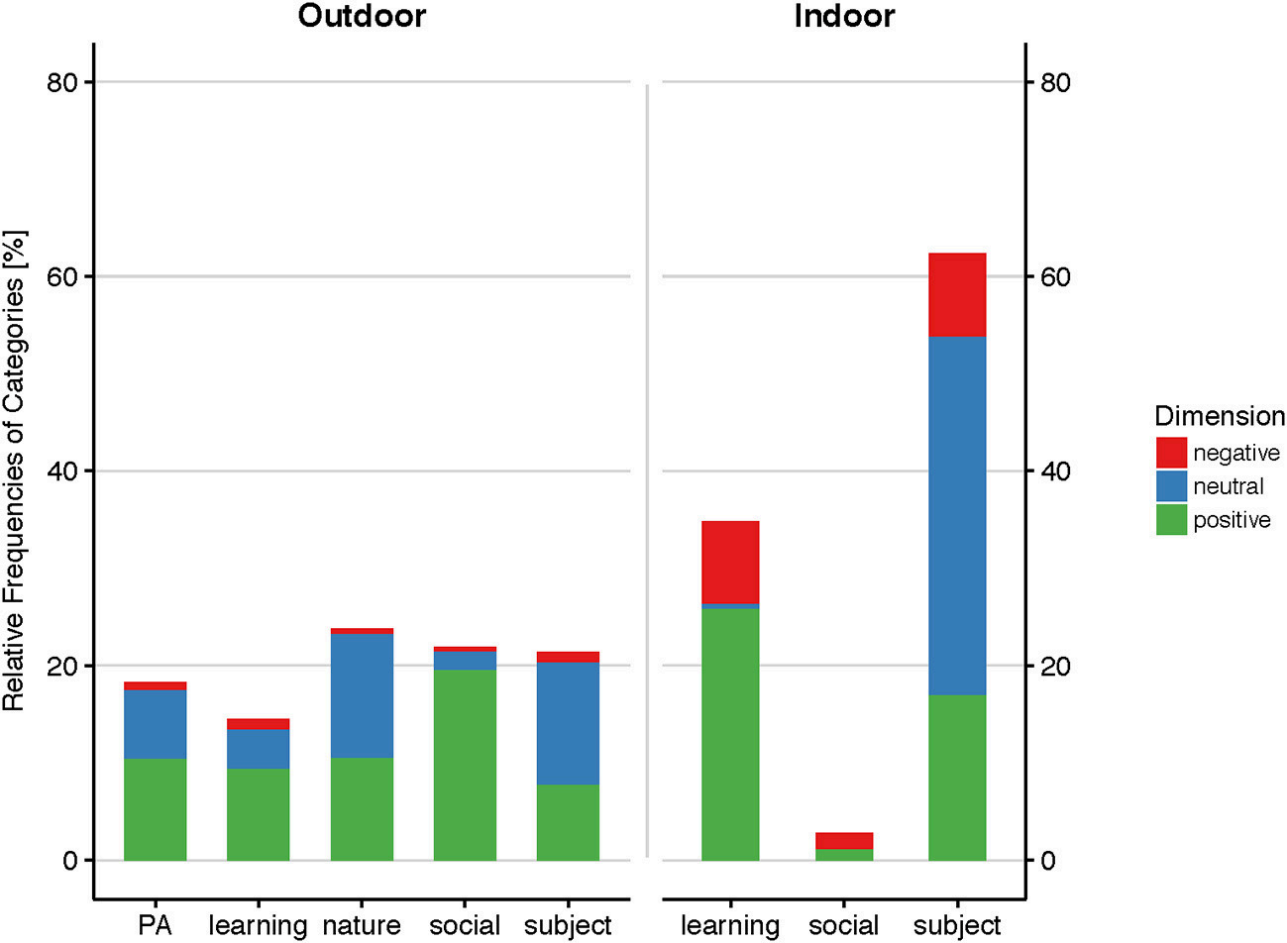
Displayed are the relative coding densities for the categories that emerged from independent coding of the text corpus for both teaching contexts. It can be seen that for the outdoor context, two more categories, “physical activity” (PA) and “nature” evolved, which indicates a richer range of experiences. Moreover, the relative frequency of negatively connoted text is considerably smaller outdoors than indoors.

For the outdoor context, 499 primary words from *n* = 180 individuals, and for the indoor context, 228 primary words from *n* = 86 individuals have been analyzed. Those have been classified by 1.385 secondary words in the outdoor- and 547 words in the indoor-context. From content analysis, five categories emerged from the outdoor context: “physical activity,” “learning,” “nature,” “social,” and “subject.” Three categories emerged from the indoor context: “learning,” “social,” and “subject” (Figure 3).

##### Subject

The category “subject” was the greatest in the class of primary and secondary words, with a density of 33%. As “subject”, all text has been coded that referred to objects, facts or things that are stand out isolated and without further association to a specific context. For the indoor context, “subject” represented 62% of all categories, and was coded 59% as “neutral,” 27% positive, and 14% negative. This distribution of dimensions is strongly skewed toward “neutral,” which accounted for 99% of all “neutral” counts in the indoor context. The overall distribution of dimensions amongst the categories was 4% negative, 38% neutral, and 58% positive, demonstrating a strong skew toward the positive. Among the 15 most frequent primary words in the indoor context, 11 were coded as “subject,” such as “botany,” “beaver,” “lung,” or “plant.” Those primary words reflect content from the curriculum of the past weeks. On the one hand, the corresponding secondary words add more information to the content: The primary word “botany,” for example, is specified by neutral secondaries as “green,” “vegetable,” “flower,” “red,” or “thin.” On the other hand, negatively coded secondaries, such as “boring,” “annoying,” “dislike,” tell more about the perception of this subject. Positive associations through secondary words often include positive attributes of those subjects. The primary word “beaver,” for example, was described as “cute,” “smart,” or “sweet” (positive secondaries).

In the outdoor context, “subject” was also coded 59% as “neutral,” however 37% as “positive” and only 4% as “negative.” The distribution of the dimensions across the five categories in the outdoor context was more diverse than in the indoor context, with 38% “neutral,” 8% negative, and 54% positive codes. Thus, the small negative proportion is comparable to that of the other categories in the outdoors, but the positive proportion was considerably smaller for subject than for the other categories in the outdoor context (cf. Figure 3). As with the indoor context, “subject” was strongly skewed toward the “neutral.” “Subject” accounted for 22% of the text, which is comparable to the indoor context, given that for outdoors, five categories emerged instead of three for the indoor context. As for the indoor context, “botany” was one of the most frequent primary words coded as “subject.” Associated “negative,” “neutral,” and “positive” secondary words were similar to the indoor context, however, “negative” connotations for “botany,” for example, were considerably less than in the indoors (3 vs. 42%). It is also interesting to note that for the outdoor context, 12 children named seven different breeds of plants using correct biological nomenclature. Not a single botanical technical term or plant name was among the indoor primaries and secondaries.

Looking at the open questions, the difference between the indoor and the outdoor contexts with respect to “subject” can be very well exemplified with the following quotes from a 12-year-old girl. The first quote was from the science class:

“*Sometimes I think, that the stuff we have learned in class is absolutely useless; on the other hand, there is stuff which is very interesting. I am looking forward to going on the excursion” (16GB11f)*.

Whereas the following quote refers to the outdoor teaching during the research week:

“*I liked it when [the teacher] explained to us what this foam at the plants means. There are animals that use that foam as protection. That's cool” (16GB11f)*.

Both quotes are referring to a subject. Indoor teaching is presented both, extremely negatively and very positively, however unspecified. The presentation of the outdoor content is framed by two short very positive emotional statements and very specifically referring to content.

##### Learning

“Learning” was the second strongest category in the indoor context, with 35% coding density. Within “indoor-learning,” 74.5% were coded “positive,” 1.5% were coded “neutral,” and 24% were coded “negative,” which resembles a bimodal distribution pattern with more negatives and more positives than expected, with a higher skew toward the positive. “Learning” refers to all words or concepts that associate with an educational process, such as “experiment,” “explorative learning,” or “presentation.” The latter three words were among the 15 most frequent primaries in the indoor context. “Explorative learning” in the indoor context was the only primary which was 100% coded “positive.” The predominant secondary words in the indoor context associated with primaries in this category were “fun” and “interesting.” The most frequent negatively associated secondaries were “boring,” “difficult,” and “unnecessary.”

For the outdoor context, “learning” accounts for 15% of the text and was considerably more associated with the neutral dimension than was the indoor context, with 65% “positive,” 28% “neutral,” and 7% “negative” codes. However, compared to the other categories in the outdoor context, negative codes are strongly overrepresented, while neutral codes are less than expected. The positive codes lie within the expected range. Predominant primary words in this category in the outdoors were “measurement” or “research,” which were associated with secondaries as “fun,” “exciting,” “great,” or “interesting.” The most frequent neutral secondary in the category “learning” was “learning,” the most frequent negative secondary is “boring.”

In the open questions, the predominant positive theme for the indoor science class was “experiments” or reports on “explorative learning” methods, such as excursions of group work, as one 12-year-old girl writes:

“*I liked it when we used the microscopes to look at the many small animals from the pond in such magnification. We have also captured one leech and have looked at it in a glass. We were allowed to do everything ourselves. That's what I like and what is cool” (16GB28f)*.

The same girl writes similarly about the outdoor class:

“*The vegetation survey was great fun, because we had to do many different things, and could also apply those things we had learned on Monday in the lab. After we had taken turns with everything, I had done everything at least once” (16GB28f)*.

Thus, active, hands-on and learning processes that are organized as group work, are associated positively, irrespective of the teaching context. Frontal teaching, exams, and lack of competence support by the teachers, are predominant negative learning experiences, most of all in the indoor context, as a 12-year-old girl writes:

“*The things we treat in science class are very interesting. Sometimes, it is really fun to learn new things. However, the teaching is very ‘boring’. Sometimes, the teacher's explanations are completely incomprehensible for me, so that I have to teach the stuff myself or ask my dad or mum. I master the stuff, but not because of the explanations during class” (15SB11f)*.

Similarly, an 11-year-old girl from the same class reports that she

“*like[d] science class which is exciting and interesting, but sometimes at the same time unclear. It would be better if we could do more experiments to the subjects in class” (15SB12f)*.

##### Social

With as little as 3%, the category “social” was only marginally represented in the indoor context, in contrast to 22% of the text in the outdoors. Moreover, 60% of all primaries and secondaries associated with the social context in the indoors were negatively connoted, with no neutral and 40% positive connotations. This results in a very strong misbalance toward the negative. Positive codes lie within the expected range compared to the other two indoor-categories, “learning” and “subject”. In the outdoors, 2% of all “social” text was negative, 8.5% neutral, and 89.5% positive, which is a very strong trend toward the positive. The negative proportion of codes lies within expectancy, whereas “neutrals” are strongly underrepresented compared to the other four outdoor-categories. Among the 15 most frequent primaries in the indoor context, only one word was from the category “social,” i.e., “fun.” Interestingly, fun has 3% negatively associated secondaries. Those refer exclusively to situations, where others are having “fun” and are “noisy” while the respondent him- or herself wants to concentrate on the teaching. Another interesting observation for the indoor context, and which represent most of the negative codes in this category, is that some students spoke detrimentally of their teachers, especially when it came to oral tests. Such teacher-complains were also found in the outdoors, but only in one case with one class, whereby an accompanying researcher was also negatively valuated in the open question section by almost half of the class. Nearly the same words have been used: He was allegedly “a little bit too rigorous,” respectively “nasty and mean.” Additionally, one accompanying student was described as a “spoilsport”—in contrast to the two other accompanying students who were “cool” and “made a lot of jokes and fun” (all quotes refer to class 16SB).

However, the general tone about the accompanying teachers, researchers, and students in the outdoors was very positive, as was text referring to the peer group. “Fun” is the most frequent secondary word in the outdoor-category “social,” followed by “cooperation” and “companionship.”

##### Nature

“Nature” is a category that emerged only in the outdoor context, with 45% positive, 53% neutral, and 2% negative expressions. This category was closely related to “subject,” showing the same misbalance of codes toward the “neutral.” The category “nature” differs from the category “subject” with respect to “experience.” The “flower” referred to in the text-book was coded as “subject,” whereas the “flower” associated with “beautiful,” “exciting,” and “interesting” as secondaries is coded as “nature.” Primary words associated with weather phenomena that had been experienced during the expedition were coded as “nature,” such as “rain,” “clouds,” “snow,” or “weather.”. “Rain” and “weather” represent most of the 2% negative connotations with “nature,” whereas “snow” (in July!) was both neutrally and positively associated, with “white” respectively “beautiful” as examples for neutral and positive secondaries. “Nature,” “weather,” “mountain,” and “glacier,” were the four predominant primaries in this category.

As one 12-year-old girl writes:

“*My best experience was the expedition because I love to be outdoors and enjoy the fresh air. There, I can think clearer. I simply love nature, because there I feel free” (16GB2f)*.

Another dominant motive in the students' expressions about nature is the “unforgettable,” “great,” or “beautiful” nature of the experience of the glacier, of summits, views or plants.

##### Physical activity (PA)

As “nature,” “physical activity” has only emerged as a category in the outdoors. Fifty-seven percent of all words coded as “PA” were positively associated, 38.5% neutrally, and 4.5% negatively, and all three dimensional coding densities lie within the expected range. Aspects of physical activity occurred during the expedition, so that “hiking” was the predominant primary concept in this category, with 63% positive, 36% neutral, and 1% negative expressions. The latter were very scarce observations of students being physically overwhelmed by the hike and thus disliking it; interestingly, we find many occurrences of students reporting about the “strenuous” hike, but associating it with “mastery” and “pride,” and they often are also associated with “nature” and “learning,” as becomes clear from the report of a 12-year-old girl:

“*When I was on the glacier, I felt my heart – literarily. I love great views, snow, and most of all, that I mastered to get up on it. Moreover, glaciers are sooooooooo interesting” (16MWB27f)*.

#### Hierarchical cluster analysis of the primary words

**Figure 4:**
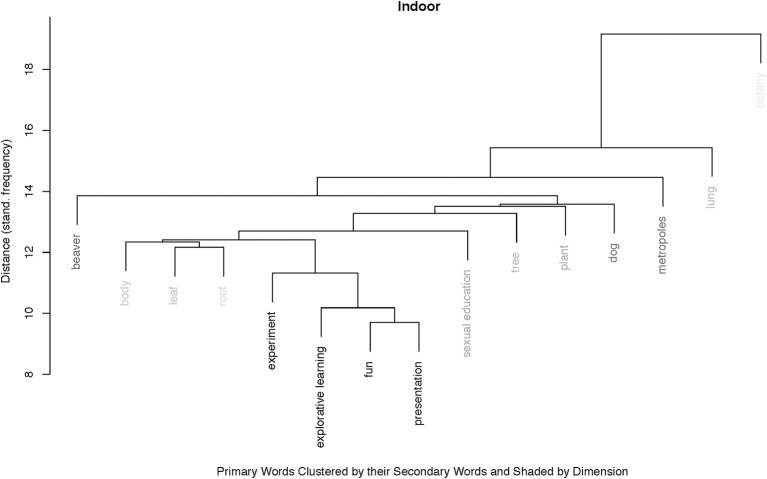
Agglomerative hierarchical cluster with the 15 most frequent primary words in the indoor context, grouped according to their secondaries. The shading indicates the percentage of positive-codes, with white indicating zero positive, and black 100% positive. We can see two main clusters: A relatively flat hierarchical cluster with 11 words, all coded as “subject” and little positive connotation, and a second cluster consisting of the four primaries, “experiment,” “explorative learning,” “fun,” and “presentation” which are all strongly positively connoted.

The analysis of the 15 most frequent primary words hierarchically clustered by their associated secondaries revealed two predominant clusters for the indoor context: One cluster, the larger one, is relatively flat consisting exclusively of primaries coded as “subject” and referring to teaching content. No specific singular secondary word defines this cluster. Their dimensional coding is rather weakly positive, which can be seen in Figure 4. The second, smaller cluster consisting only of “experiment,” “explorative learning,” “fun,” and “presentation” shows an extremely strong positive connotation and represents words from the categories “learning” and “social.” “Fun” is also the predominant secondary word for this sub-cluster.

**Figure 5:**
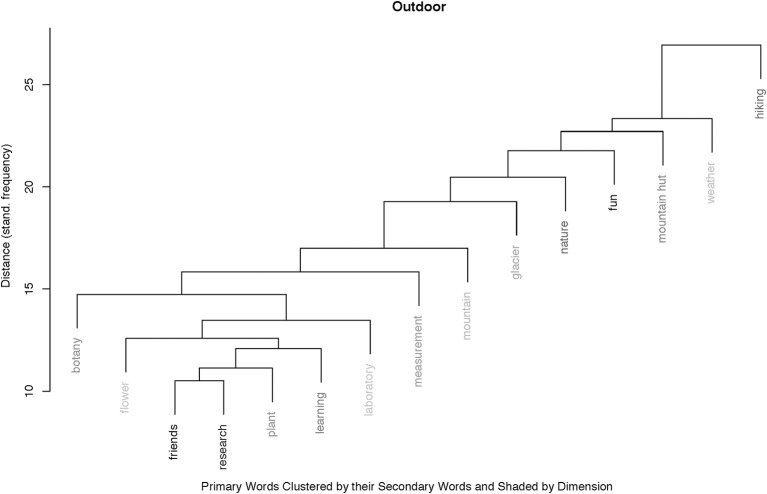
Agglomerative hierarchical cluster with the 15 most frequent primary words in the outdoor context, grouped according to their secondaries. The shading indicates the percentage of positive-codes, with white indicating zero positive, and black 100% positive. We can see two main clusters: A linear hierarchical cluster with 7 words, all elements of the “hiking,” and a second cluster consisting of eight primaries, hinting at research activities. “Friends” and “research” make up an interesting mirco-cluster, indicating the perceived peer-relatedness during the outdoor research tasks.

The cluster for the outdoor context (Figure 5), reveals a completely different pattern: it consists also of two main clusters of about the same size. The first cluster shows a liner hierarchical structure from “hiking” down to “mountain,” with primaries from “physical activity,” “social,” and “nature,” grouping concepts from the expedition quite loosely, without any specific outstanding secondary word. The second cluster is relatively flat, consisting of primaries being coded in the categories “learning” and “social.” The two primaries “friends” and “research” define a rather noticeable micro-cluster within the second outdoor-cluster: both are strongly positively coded. The predominant secondary word, which defines the distance in this cluster, is “fun.”

**Figure 6:**
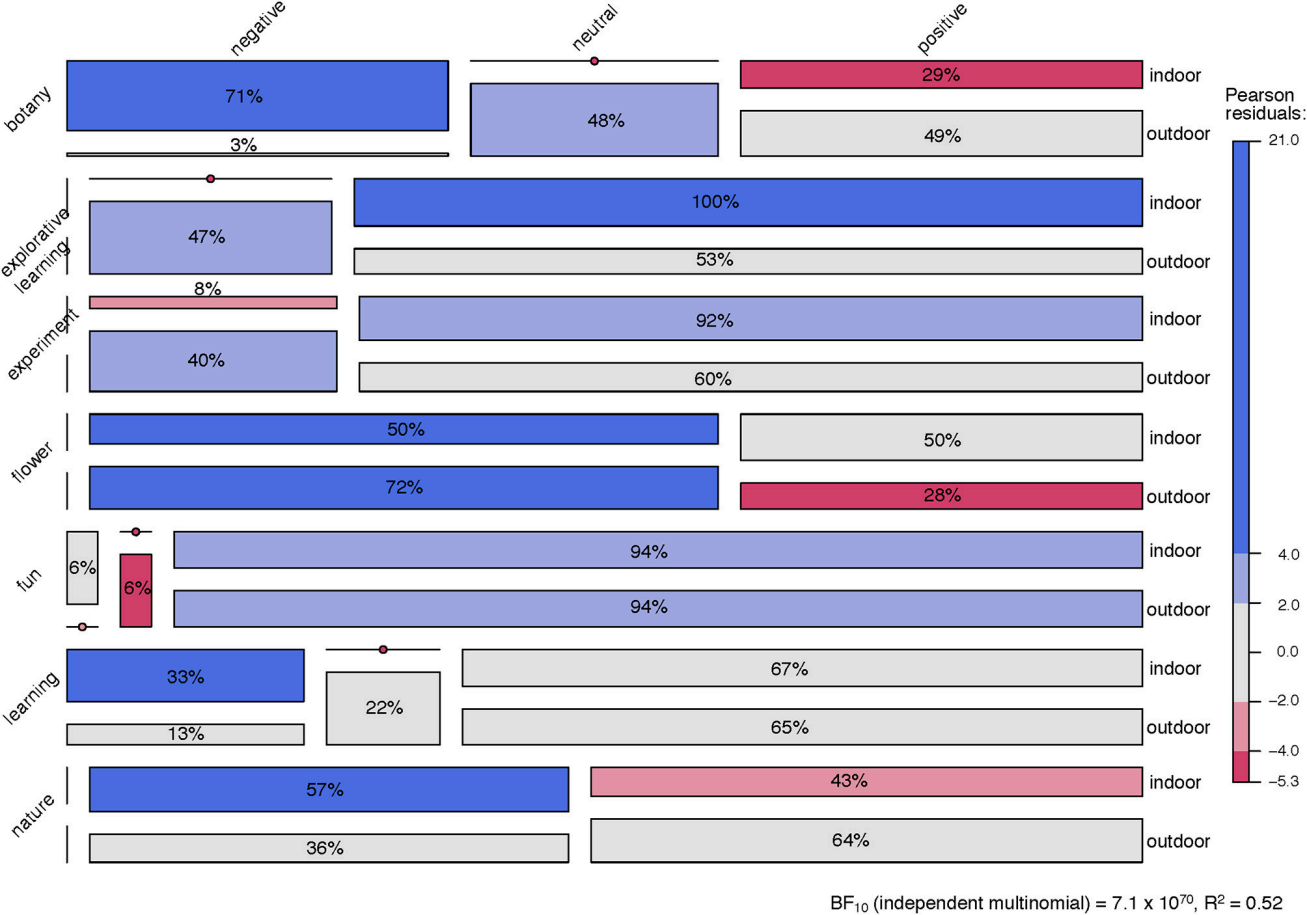
Mosaic plot of seven intersecting primaries that belong to the set of the 15 most frequent primaries, either in the indoor or the outdoor contexts. The tiles represent the cells of the underlying 7 (primaries) × 3 (dimensions) cross table, with tile-sizes referring to the cell-values, here: percentiles. Vertical or horizontal lines indicate a cell-value of 0%; dots on those lines refer to the respective Pearson residuals. The latter are displayed in color-shading in order to indicate the grade of deviation from expected values within the respective dimensions (“columns”).

As can be seen from Figures 4, 5, there were seven primaries among the 15 most frequent that occurred in both contexts, i.e., “botany,” “explorative learning,” “experiment,” “flower,” “fun,” “learning,” and “nature.” They are displayed in Figure 6 in relation to the relative distribution notated in percentiles of their dimensional coding, for both teaching contexts. This mosaic plot, which is basically a graphical display of the underlying seven primaries by three dimensions cross-table, allows to compare the usage of the primaries in each context with respect to the statistically expected relative frequency of occurrence.

“Botany” was the most frequent in the indoor context and coded 71% “negative” vs. 3% negative in the outdoors, which is a strong skew toward negative for the indoors. The few negative codes for botany in the outdoors were, however, fully in the expectancy range compared to the dimensions of the other six intersecting primaries. Accordingly, “botany” was coded 29% positive indoors vs. 49% positive outdoors, which is less than expected in the indoors, but fully in the expected range for the outdoors.

Interestingly, in the indoors, “flower” was more positively perceived (with 50%) than was “botany.” The other 50% of the codes were “neutral.” In the outdoors, with 28%, “flower” was less positively connoted than for the indoors, and was also less positively connoted than expected. In neither context was “flower” rated negatively.

As already reported above, “explorative learning” in the indoor context was, with 100% “positive,” the most unambiguous primary in the data set. With 47% neutral vs. 53% positive codes in the outdoor context, “explorative learning” was fully in the expectancy range for the positive, and slightly above this range for the neutral codes—resulting from zero negative codes for this primary.

As “explorative learning,” “experiment” was with 92% highly positively connoted in the indoor context vs. 60% in the outdoors. Again, this primary had zero “negatives” in either context, however, with only 8% “neutrals” in the indoors vs. 40% in the outdoors, one can see a moderately strong skew toward the positive in the indoors, whereas there was a moderate leaning toward the “neutral” in the outdoors.

“Fun” was, in both contexts, with 94% each, rated exceedingly and not surprisingly “positive.” However, it is surprising, as noted above, that in the indoor context, 6% of all ratings for “fun” were “negative.”

“Learning” was, apart from a slight negative tendency in the indoors, fully within the expected range of relative frequencies.

“Nature” instead showed, with a distribution of 57% neutral vs. 43% positive codes in the indoors a clear tendency toward the neural. In the outdoor context, on the contrary, “nature” was perceived as could have been expected from the distribution pattern with respect to the other six intersecting primaries. Also with “nature,” no negative primary could be found in the text body.

## Contextualization and discussion

### Self-determination in science class and basic psychological needs

The main result with the SDI analyses was that autonomy- and competence- support have the biggest effect on the student's motivational behavior, independent from the teaching context. A similar finding was reported by Tsai et al. (2008), who found that perceived autonomy support during lessons predicted students' interest experience in the classroom. The context-invariance is suggested by Self-Determination Theory (Deci and Ryan, 2000) and indirectly validates the applied measurement instruments.

Contextualizing this finding with the qualitative data, experiences of competence and autonomy are, in both contexts, strongly related to explorative learning through making use of experiments or survey-methods. Furthermore, traditional “from the front” teaching and excessive teacher-explanations can have a negative effect on competence and autonomy experiences.

Girls show, in both contexts, a more intrinsic learning motivational behavior in science compared to boys. This finding is in contrast to previous research which reports that boys are to a higher degree motivated to learn in science and do experiments, than girls (Mézes and Erb, 2013). However, it should be noted that Mézes and Erb's study focused on experiments in physics, while during the research weeks of the current study, botanical, chemical, and physical experiments were embedded in a biological research question (Dettweiler et al., 2015b). The girls' higher SDI values for the outdoor teaching might be explained by free-choice-experiments which have shown that girls prefer biology, while boys prefer physics (Baram-Tsabari and Yarden, 2008). The girls' higher indoor values can be explained by the matter of fact that the curriculum for most of the children participating in this study focused mostly on biology during the period immediately prior to the research weeks. This can also be seen from the primary words for the indoor context, where 9 of 10 subject-related words are biological terms.

The finding that learning, irrespective of gender, is more self-regulated in the outdoors can be explained with the consequent use of explorative and experiential teaching strategies, for which higher situational interest of children in our age group has been reported (Brandt, 2005; Thomas and Müller, 2014; Dettweiler et al., 2015a; Cahyono et al., 2016). As we have seen, the “freedom” perceived in teaching situations, both during the intervention and during occasional learning sessions outside the classroom during “normal” science class at the schools, is highly valued by the students. This can also be seen in the cluster plots where learning strategies that support the students' autonomy, as presentations on self-chosen topics or experiments with an explorative learning approach, are associated with “fun” (see Figure 5).

Student-teacher relations have a relatively smaller but still very high impact on the students' motivational behavior. Additionally, we can see from the examples quoted, that those teachers who literarily go the extra mile in science class and take their students in science class out on excursions etc., are liked the most by students. Student-teacher-relatedness is tightly connected to the students' perceived autonomy- and competence-support by a given teacher, as taking students out facilitates their experiences of competence- and autonomy-support. This has also been shown in a study by Lopes et al. (2011), who reported that the effects of learning experiences provided to students are strongly determined by the teacher-student relations, and that the quality of those experiences influence the development of student-competences. Moreover, social support from teachers, peers and parents has a direct effect on math and science perceived abilities and an indirect effect mediated through math and science attitudes (Rice et al., 2013). This is very much in accordance with the current findings comparing a teacher-centered approach with experiential teaching strategies. While a teacher-centered approach provides obviously higher short-term learning success, regular hands-on instruction can lead to sustainable learning effects (Gerstner and Bogner, 2010).

Contextualizing all findings with literature, we can say that occasional but regular practical (outdoor) learning sessions encourage students' interest in science by fostering autonomy, self-perceived competence, and student-teacher relatedness, and they contribute to sustain their interest in science. However, we cannot say anything about objective competence growth in science through outdoor learning, and literature questions the effectiveness of such practical work in science class with respect to the students' competences (Abrahams and Millar, 2008).

That in the current study student-student relations seem to be irrelevant for the students' motivational behavior also indicates more about the children's obviously stable social relations that are largely unaffected by school. Nevertheless, we learn from the textual data that relations with their peers are important for the students. The cluster analysis for the outdoor context reveals that “research” goes well with “friends.”

### Elevated SDI outdoors compared to indoors: potential mediators

The Bayesian linear mixed model reveals that the difference in perceived competence support can best explain the difference in self-regulated motivational behavior. The examples quoted above with respect to explorative, experiential and hands-on learning, a teaching concept which is simply much more dominant in the outdoor context than in the indoors, are unequivocal. However, the diffC-Model only explains about 15% of the variance in the data. This means that 85% are not explained with increased competence support. By exemplarily looking at the conception of “botany,” the substantial difference between the indoor and outdoor contexts becomes clearer:

As reported above, “botany” was the most frequent primary in the indoor context, and also the most negatively associated one. It clusters with long distance to the next primary, “lung,” which has only to do with “botany” as “lung” is another curriculum-content and a “subject.” Suitably, in this above described cluster of “subjects” in the indoor teaching context, the other four botanical primaries, “leaf,” “root,” “tree,” and “plant” are clustered in three different micro-clusters, with “leaf” and “root” being closer connected to “body” or “sexual education” than with “tree” or “plant”. This demonstrates well the lack of perceived connectivity of topics in the indoor context. However, the micro-cluster with “leaf” and “root” is tightly connected with the micro-cluster “explorative learning” with “fun” in the center. From the above reported analysis of the corresponding secondaries and the open questions we can see that those primaries stem from situations of short learning sessions outside the classroom or from self-chosen presentation topics, which are associated with fun, also for the “indoor” context. Thus, we can say that topics taught in the indoor context are perceived disconnected, and whenever explorative learning methods are applied, a positive connection with curriculum subjects can be made.

This is even more obvious for the outdoor context. That 12 students used the correct botanical nomenclature when speaking of “plants” or “flowers,” and recall together seven different breeds, is noteworthy in so far as this was a free association test. They had not been asked anything specific about curriculum content. By looking at the cluster plot for the outdoor primaries, it becomes clear that the three most frequent botanical primaries, “plant,” “flower,” and “botany,” are grouped together with “learning,” “measurement,” “laboratory,” “research,” and “friends,” with the latter two making up a micro-cluster. This integration of the botanical terms in the “research-cluster” displays an active and intact learning culture during the outdoor teaching.

Another driving factor in this cluster is the secondary word “fun” which is, as described above, the dominant secondary clustering those primaries. “Fun” is an ingredient in educational settings that is often labeled as “situational interest” in “quantitative” literature on science teaching (Hidi and Renninger, 2006; Tsai et al., 2008), and is not further specified. However, a recent Australian mixed-methods study addressed this deficit and conduced focus-interviews with a sub-set of 25 students of the total group of pre-service elementary teachers who were enrolled in a science course (*n* = 229) to identify “underlying causes of situational interest.” They found that “teaching techniques that generated situational interest included hands-on activities, personal anecdotes, fun facts, demonstrations of science toys, science magic, clear explanations, and the use of models and artifacts” (Palmer et al., 2016). A qualitative Turkish study searched into “enriched educational practices” in science class with 7th graders. The practices included explorative teaching methods, problem-based learning, teaching of relevant subjects by experts, and teaching of subjects in a science center. The evaluation revealed that the students “found the science course more fun, more effective, interesting and good, and they liked the course more and understood and learned the subjects in the course as a result of the enriched educational practices carried out in science courses” (Idin and Aydogdu, 2016). The importance of positive affection, with the subcategories fun and enjoyment, for motivational behavior has been theoretically described by e.g., Aspinwall (1998). An experimental control group design study by Isen and Reeve (2005) revealed that if participants where in a positive affect condition and could freely choose among activities, they preferred enjoyable tasks but also successfully completed rather interesting work that needed to be done. Therefore, positive affect can lead to forward looking thinking, self-control and intrinsic motivation. Moreover, the matter of fact that two more categories, “nature” and “physical activity” emerged from the analysis of the outdoor text corpus, indicates that teaching during the research weeks can be considered as more “enriched” than the regular indoor classes. “Fun” and “freedom” play a dominant role during in the secondary words associated with “hiking,” together with feelings of achievement and mastery. This might contribute to the grade of the students' perceived competence and autonomy, which should not be underestimated. The pilot study on this program from 2014 had revealed that group dynamics and physical activity levels are relevant factors for positive motivational behavior and that “fun” is one of the driving factors (Dettweiler et al., 2015a). Similarly, physical activity and social interaction have been reported as “minor sources of situational interest” by Palmer et al. (2016).

Another enriching factor in the outdoors is “nature.” In our generated body of text, many overwhelming and extraordinary experiences of the beauty of nature can be found. The “expedition” provides the students with completely new and “unforgettable” experiences. This strong emotional impact of nature experiences on the students might be a limiting factor in generalizing results of the students' outdoor-teaching experience for the school context, since it is rather improbable that all school children can experience such a residential course during their school careers. However, with curriculum reforms that react on the tremendous global challenges, such as climate change, many such hand-on science teaching programs have emerged. To address this call to action, K-12 Science education in the United States, for example, is undergoing a period of transition from a disconnected fact-based system to a holistic approach integrating scientific practices, crosscutting concepts that span across the scientific disciplines, and the application of core science content to real-world scenarios. This transformative vision is laid out in detail by the National Academy of Sciences in both the Framework for K-12 Science Education (National Research Council, 2012) and the Next Generation Science Standards (Ngss Lead States, 2013). The program “PlantingScience” in Canada and the USA, for example, provides similar to the “research weeks,” “scientists, resources, and activities to support innovation in teaching, learning, and mentoring for student-centered outdoor plant investigations […] that integrate scientific practices and big ideas in biology” (PlantingScience, 2017). The program is explicitly designed to meet the guidelines in the Next Generation Science Standards and other twenty first century education standards. A study by Stephen Scogin, who evaluated this botanical online resource to teachers and schools, found that

*[t]eachers contributed to student motivation by giving students more freedom, challenging students to take projects deeper, encouraging, and scaffolding. Scientists contributed to student motivation by providing explanations, asking questions, encouraging, and offering themselves as partners in the inquiry process. Several positive student outcomes of the program were uncovered and included increased positivity, greater willingness to take projects deeper, better understanding of scientific concepts, and greater commitments to collaboration. (Scogin*, *2016**)*.

This corresponds well with findings from a study in Wales, where college students were taken out on a quite similar residential field-trip under the supervision of staff from an external scientific institution. The authors report that

*[s] tudent feedback resulting from the intervention showed impressive levels of improvement in general appreciation of the course. Students also suggested that they would have liked a longer module, despite the intensive workload they experienced. All areas explored (general knowledge, research-based evaluation criteria, group and individual work) showed improvements in student evaluation after the module. We conclude that a residential, integrated experience of scientific research […] can produce significant positive, active learning experiences to the students (Gamarra et al., 2010)*.

If we cannot say something about the effectiveness of such programs with respect to the students' performance in science, it is important that the students enjoy science, and that they come to understand the “nature” of science, e.g., through practical field work.

### Limitations

The generalizability of the reported findings might justifiably be questioned. As it is a common problem in educational research on specific interventions, the results have always to be understood in the perspective of this specific program, which (a) shows a certain variability from class to class, with respect to weather conditions, the accompanying teachers, students, staff, and randomization not being realizable due to the classroom setting. There are many factors that cannot be controlled, so that (b) it cannot be sensibly conceived as “repeatable.” Some prominent researchers would go as far as to refuse the language of “evidence” for such studies and focus more on value-based educational research (Biesta, 2010). We are trying to address this important critique on contemporary educational research with our mixed-methods design, balancing out generalizable factual statements and in-depth-understanding, using the Bayesian approach. The comparability to other studies using “similar” teaching approaches can only be justified with reference to more or less well-defined general concepts such as “explorative learning,” “hands-on learning,” “experiments” etc., which could be called, in Wittgensteinian terms, “family similarities.” However, this is a very general and deep-reaching epistemological problem which can best be addressed with the internal realist view we endorse in this survey and which is described and argued for in more detail by Dettweiler (2015).

Moreover, due to summer holidays shortly after the research weeks, it was not possible to add a sensible follow-up survey after a certain period of time, say 6 weeks after the intervention. Thus, we cannot say anything about the sustaining factor of such learning strategies on the students' learning motivational behavior in the indoor classes. Further research, using a full prospective empirical design, should address this gap in educational research. With respect to the effects of regular and compulsory outdoor teaching on the students' motivational behavior, a study performed on *n* = 834 Danish children aged 9–13 and whose design has been recently published, will hopefully close some gaps (Nielsen et al., 2016).

The reported gender-bias in our target group, which results from the inclusion of one girls-only class, does also result in a higher density of female anchor-quotes, which can be seen as both, a result and a limitation. The girls' quotes were more to the point than the boys', and, of course, there were simply more to choose from. But since the diffSDI-Model suggests that the increase of motivational behavior can best be explained with gender-invariant student behavior, it would have been non-advantageous to exclude this girls-only class from the data set. To this point it is also interesting that the current findings are consistent with those of other studies in this area.

## Conclusion

The contextualization of the results from the various data on the student's motivational behavior with respect to the satisfaction of basic psychological needs, and the discussion with recent literature, has shown that outdoor residential programs using explorative learning methodology can drastically improve the student's learning attitudes. However, as we have shown above, much of those dynamics, which can develop a strong thrust toward “situational interest” and “learning motivation” in the outdoors during residential programs, can also be achieved in occasional short excursions supplementary to the regular science curriculum. The main message of this analysis of a residential outdoor science teaching program is to use the teaching techniques explored and developed for this and other similar residential programs and include those occasionally but regularly in the normal school curriculum; the positive effects of such regular teaching sessions outside the classroom have only recently been analyzed and described (Becker et al., 2017).

Be it on residential programs or as an integrated part of the normal science curriculum, or both, there is very strong evidence that teaching science outdoors on a regular basis is an appropriate strategy to meet the challenges of the twenty first century, and might also be a solution to bridge the still-existing gap between science teaching and environmental education (Wals et al., 2014).

## Ethics statement

This study was carried out in accordance with the German legal regulations with written informed consent from all subjects. All subjects as well as their parents gave written informed consent in accordance with the Declaration of Helsinki. The protocol was approved by the relevant school authorities.

## Author contributions

UD conceived and designed the study; CB and GL collected the data; UD, GL, CB, and PS analyzed the data; UD wrote most of the paper with substantial contributions from all other authors. All authors proved the final version of the manuscript.

### Conflict of interest statement

The authors declare that the research was conducted in the absence of any commercial or financial relationships that could be construed as a potential conflict of interest.

## Footnotes

^1^The code for the student quotes reads as follows: the two digits indicate the year (14-16), the following capital letters encode the school, the next digit(s) indicate the running number identifying the individual respondents, and the last minuscule encodes the respondents' gender (“m” for “male” and “f” for “female”).

^2^The codes for the interview respondents do not include definite identifiers since they preferred to not have connected their interview responses to the data set.

## References

[B1] Abrahams I. Millar R. (2008). Does practical work really work? A study of the effectiveness of practical work as a teaching and learning method in school science. Int. J. Sci. Educ. 30, 1945–1969. 10.1080/09500690701749305

[B2] Aspinwall L. G. (1998). Rethinking the role of positive affect in self-regulation. Motiv. Emot. 22, 1–32. 10.1023/A:1023080224401

[B3] Baram-Tsabari A. Yarden A. (2008). Girls' biology, boys' physics: evidence from free-choice science learning settings. Res. Sci. Technol. Educ. 26, 75–92. 10.1080/02635140701847538

[B4] Becker C. Lauterbach G. Spengler S. Dettweiler U. Mess F. (2017). Effects of regular classes in outdoor education settings: a systematic review on students' learning, social and health dimensions. Int. J. Environ. Res. Public Health 14:485. 10.3390/ijerph14050485 28475167 PMC5451936

[B5] Biesta G. J. J. (2010). Why “What Works” Still Won't Work: from evidence-based education to value-based education. Stud. Philos. Educ. 29, 491–503. 10.1007/s11217-010-9191-x

[B6] Brandt A. (2005). Förderung von Motivation und Interesse durch außerschulische Experimentierlabors [Enhancing motivation and interest through extra-school laboratories]. Göttingen: Cuvillier.

[B7] Cahyono A. Haryanto S. Sudarsono (2016). Increasing motivation and science learning achievement through the implementation of outdoor cooperative learning model in class VIII SMP 2 Banguntapan Academic Year 2015/2016. J. Educ. Pract. 7, 21–26.

[B8] Corpus J. H. Mcclintic-Gilbert M. S. Hayenga A. O. (2009). Within-year changes in children's intrinsic and extrinsic motivational orientations: contextual predictors and academic outcomes. Contemp. Educ. Psychol. 34, 154–166. 10.1016/j.cedpsych.2009.01.001

[B9] de Andrade J. C. De Aguiar Sobral L. Ares G. Deliza R. (2016). Understanding consumers' perception of lamb meat using free word association. Meat Sci. 117, 68–74. 10.1016/j.meatsci.2016.02.039 26946479

[B10] De Finetti B. (1974). Theory of Probability. A Critical Indtroductory Treatment. London; New York, NY, Sydney, NSW; Toronto, ON: John Wiley & Sons.

[B11] De Finetti B. (1975). Theory of Probability. A Critical Indtroductory Treatment. London; New York, NY: Sydney, NSW; Toronto, ON: John Wiley & Sons.

[B12] DeCharms R. (1968). Personal Causation. New York, NY: Academic Press.

[B13] Deci E. L. Ryan R. M. (2000). The “what” and “why” of goal pursuits: human needs and the self-determination of behavior. Psychol. Inq. 11, 227–268. 10.1207/S15327965PLI1104_01

[B14] Deci E. L. Vansteenkiste M. (2004). Self-determination theory and basic need satisfaction: understanding human development in positive psychology. Ricerche di Psichologia 27, 17–34.

[B15] Dettweiler U. (2015). Educational Research in the Mirror of Nature. Theoretical, Epistemological, and Empirical Aspects of Mixed-Method Approaches in Outdoor Education. PhD Thesis, Technische Universität München.

[B16] Dettweiler U. Ünlü A. (2015). Testing the reliability and validity of a reduced Academic Self-Regulation Questionnaire (SRQ-A) in a mixed-methods approach, in Eighth SELF Biennial International Conference (Kiel).

[B17] Dettweiler U. Lauterbach G. Becker C. Ünlü A. Gschrey B. (2015a). Investigating the motivational behaviour of pupils during outdoor science teaching within self-determination theory. Front. Psychol. 6:125. 10.3389/fpsyg.2015.00125 25741301 PMC4331641

[B18] Dettweiler U. Lauterbach G. Mayer B. Menzel A. (2015b). The space-for-time approach as a didactical tool. Modeling climate change during an Alpine educational research expedition, in 8th World Environmental Education Congress (Göteborg).

[B19] Gamarra J. G. P. Ironside J. E. De Vere N. Allainguillaume J. Wilkinson M. J. (2010). Research-based residential fieldwork learning: double bonus? Biosci. Educ. 16, 1–9. 10.3108/beej.16.6

[B20] Gerstner S. Bogner F. X. (2010). Cognitive achievement and motivation in hands-on and teacher-centred science classes: does an additional hands-on consolidation phase (concept mapping) optimise cognitive learning at work stations? Int. J. Sci. Educ. 32, 849–870. 10.1080/09500690902803604

[B21] Gnambs T. Hanfstingl B. (2016). The decline of academic motivation during adolescence: an accelerated longitudinal cohort analysis on the effect of psychological need satisfaction. Educ. Psychol. 36, 1691–1705. 10.1080/01443410.2015.1113236

[B22] Gottfried A. E. Fleming J. S. Gottfried A. W. (2001). Continuity of academic intrinsic motivation from childhood through late adolescence: a longitudinal study. J. Educ. Psychol. 93, 3–13. 10.1037/0022-0663.93.1.3

[B23] Groemping U. (2006). Relative importance for linear regression in R: the package relaimpo. J. Stat. Softw. 17, 1–27. 10.18637/jss.v017.i01

[B24] Hacking I. (1983). Representing and Intervening: Introductory Topics in the Philosophy of Natural Science. Cambridge: Cambridge University Press.

[B25] Hacking I. (2001). An Introduction to Probability and Inductive Logic Cambridge. Cambridge: Cambridge University Press.

[B26] Hidi S. Renninger K. A. (2006). The four-phase model of interest development. Educ. Psychol. 41, 111–127. 10.1207/s15326985ep4102_4

[B27] Humphreys M. Jacobs A. M. (2015). Mixing methods: a bayesian approach. Am. Polit. Sci. Rev. 109, 653–673. 10.1017/S0003055415000453

[B28] Idin S. Aydogdu C. (2016). Opinions of 7th grade students about enriched educational practices in the scope of science course. Int. J. Res. Educ. Sci. 2, 345–358. 10.21890/ijres.17681

[B29] Isen A. M. Reeve J. (2005). The influence of positive affect on intrinsic and extrinsic motivation: facilitating enjoyment of play, responsible work behavior, and self-control. Motiv. Emot. 29, 295–323. 10.1007/s11031-006-9019-8

[B30] Jeffrey R. C. (2004). Subjective Probability: The Real Thing. Cambridge; New York, NY: Cambridge University Press.

[B31] Jeffreys H. (1961). Theeory of Probability. Oxford: Oxford University Press.

[B32] Johnson R. B. Onwuegbuzie A. J. (2004). Mixed methods research: a research paradigm whose time has come. Educ. Res. 33, 14–26. 10.3102/0013189X033007014

[B33] Lee M. D. Wagenmakers E.-J. (2013). Bayesian Cognitive Modeling: A Practical Course. Cambridge; New York, NY: Cambridge University Press.

[B34] Levesque C. Zuehlke A. N. Stanek L. R. Ryan R. M. (2004). Autonomy and competence in German and American university students: a comparative study based on the self-determination-theory. J. Educ. Psychol. 96, 68–84. 10.1037/0022-0663.96.1.68

[B35] Lopes J. B. Branco J. Jimenez-Aleixandre M. P. (2011). “Learning Experience” provided by science teaching practice in a classroom and the development of students' competences. Res. Sci. Educ. 41, 787–809. 10.1007/s11165-010-9190-5

[B36] Ly A. Verhagen J. Wagenmakers E. J. (2016). Harold Jeffreys's default Bayes factor hypothesis tests: explanation, extension, and application in psychology. J. Math. Psychol. 72, 19–32. 10.1016/j.jmp.2015.06.004

[B37] Maechler M. Rousseeuw P. Struyf A. Hubert M. Hornik K. (2016). cluster: Cluster Analysis Basics and Extensions. R Package Version 2.0.5. Vienna: R Foundation.

[B38] Mayring P. (2014). Qualitative Content Analysis: Theoretical Foundation, Basic Procedures and Software Solution. Klagenfurt: Beltz.

[B39] Mézes C. Erb R. (2013). Zur Motivation beim Experimentieren, in Inquiry-based Learning - Forschendes Lernen, (2013) S, ed Bernholt S. (Kiel: IPN-Verlag), 86–88.

[B40] Morey R. D. Rouder J. N. (2015). BayesFactor: Computation of Bayes Factors for Common Designs. R package version 0.9.12–2. Vienna: R Foundation.

[B41] Müller F. Hanfstingl B. Andreitz I. (2007). ”Skalen zur motivationalen Regulation beim Lernen von Schülerinnen und Schülern. Adaptierte und ergänzte Version des Academic Self-Regulation Questionnaire (SRQ-A) nach Ryan & Connell [Scales of motivational learning regulation of pupils. Adapted and extended version of the Academic Self-Regulation Questionnarie (SRQ-A) of Ryan & Connell],“ in Institut Für Unterrichts- Und Schulentwicklung ed Beiträge W. (Klagenfurt: Alpen-Adria-Universität), 1–34.

[B42] National Research Council (2012). Education for Life and Work: Developing Transferable Knowledge and Skills in the 21st Century. Washington, DC: The National Academies Press.

[B43] Ngss Lead States (2013). Next Generation Science Standards: For States, by States. Washington DC: The National Academies Press.

[B44] Nielsen G. Mygind E. Bølling M. Otte C. R. Schneller M. B. Schipperijn J.. (2016). A quasi-experimental cross-disciplinary evaluation of the impacts of education outside the classroom on pupils' physical activity, well-being and learning: the TEACHOUT study protocol. BMC Public Health 16:1117. 10.1186/s12889-016-3780-8 27776502 PMC5078947

[B45] Niemiec C. P. Ryan R. M. (2009). Autonomy, competence, and relatedness in the classroom. School Field 7, 133–144. 10.1177/1477878509104318

[B46] Oevermann U. (1979). Die Methodologie einer “objektiven Hermeneutik” und ihre allgemeine forschungslogische Bedeutung in den Sozialwissenschaften, in Interpretative Verfahren in den Sozial- und Textwissenschaften, ed. Soeffner H.-G. (Stuttgart: Metzler), 352–434.

[B47] Palmer D. H. Dixon J. Archer J. (2016). Identifying Underlying Causes of Situational Interest in a Science Course for Preservice Elementary Teachers. Sci. Educ. 100, 1039–1061. 10.1002/sce.21244

[B48] PlantingScience (2017). Available online at: https://www.plantingscience.org/about/pscanada (Accessed June 2, 2017).

[B49] Putnam H. (1981). Reason, Truth and History. Cambridge: Cambridge University Press.

[B50] Putnam H. (1988). Representation and Reality. Cambridge, MA; London: MIT Press.

[B51] R Development Core Team (2008). R: A Language and Environment for Statistical Computing [Computer software]. Vienna: R Foundation for Statistical Computing.

[B52] Reeve J. (2009). Why teachers adopt a controlling motivating style toward students and how they can become more autonomy supportive. Educ. Psychol. 44, 159–175. 10.1080/00461520903028990

[B53] Reeve J. Jang H. Carrell D. Jeon S. Barch J. (2004). Enhancing students' engagement by increasing teachers' autonomy support. Motiv. Emot. 28, 147–169. 10.1023/B:MOEM.0000032312.95499.6f

[B54] Rice L. Barth J. M. Guadagno R. E. Smith G. P. Mccallum D. M. Asert (2013). The role of social support in students' perceived abilities and attitudes toward math and science. J. Youth Adolesc. 42, 1028–1040. 10.1007/s10964-012-9801-8 22890901

[B55] Roth G. Assor A. Kanat-Maymon Y. Kaplan H. (2007). Autonomous motivation for teaching: how self-determined teaching may lead to self-determined learning. J. Educ. Psychol. 99, 761–774. 10.1037/0022-0663.99.4.761

[B56] Rouder J. N. Morey R. D. (2012). Default bayes factors for model selection in regression. Multivariate Behav. Res. 47, 877–903. 10.1080/00273171.2012.734737 26735007

[B57] Ryan R. M. Connell J. P. (1989). Perceived locus of causality and internalization: examining reasons for acting in two domains. J. Pers. Soc. Psychol. 57, 749–761. 10.1037/0022-3514.57.5.749 2810024

[B58] Saint-Mont U. (2011). Statistik im Forschungsprozess. Eine Philosophie der Statistik als Baustein einer integrativen Wissenschaftstheorie. Heidelberg: Physica-Verlag.

[B59] Scogin S. C. (2016). Identifying the factors leading to success: how an innovative science curriculum cultivates student motivation. J. Sci. Educ. Technol. 25, 375–393. 10.1007/s10956-015-9600-6

[B60] Scrutton R. Beames S. (2015). Measuring the unmeasurable: upholding rigor in quantitative studies of personal and social development in outdoor adventure education. J. Exp. Educ. 38, 8–25. 10.1177/1053825913514730

[B61] Sierens E. Vansteenkiste M. Goossens L. Soenens B. Dochy F. (2009). The synergistic relationship of perceived autonomy support and structure in the prediction of self-regulated learning. Br. J. Educ. Psychol. 79, 57–68. 10.1348/000709908X304398 18466671

[B62] Sproule J. Martindale R. Wang J. Allison P. Nash C. Gray S. (2013). Investigating the experience of outdoor and adventurous project work in an educational setting using a self-determination framework. Euro. Phys. Educ. Rev. 19, 315–328. 10.1177/1356336X13495629

[B63] Thomas A. E. Müller F. H. (2014). Autonomy support: a key for understanding students learning motivation in science? Zeitschrift für Bildungsforschung 4, 43–61. 10.1007/s35834-013-0073-5

[B64] Tsai Y.-M. Kunter M. Lüdtke O. Trautwein U. Ryan R. M. (2008). What makes lessons interesting? the role of situational and individual factors in three school subjects. J. Educ. Psychol. 100, 460–472. 10.1037/0022-0663.100.2.460

[B65] Ünlü A. Dettweiler U. (2015). Motivation internalization and simplex structure in Self-Determination theory. Psychol. Rep. 117, 675–691. 10.2466/14.PR0.117c25z1 26595290

[B66] Vallerand R. J. Fortier M. S. Guay F. (1997). Self-determination and persistence in a real-life setting: toward a motivational model of high school dropout. J. Pers. Soc. Psychol. 72, 1161–1176. 10.1037/0022-3514.72.5.1161 9150590

[B67] Vansteenkiste M. Sierens E. Soenens B. Luyckx K. Lens W. (2009). Motivational profiles from a self-determination perspective: the quality of motivation matters. J. Educ. Psychol. 101, 671–688. 10.1037/a0015083

[B68] Wals A. E. Brody M. Dillon J. Stevenson R. B. (2014). Convergence between science and environmental education. Science 344, 583–584. 10.1126/science.1250515 24812386

[B69] Wang C. K. J. Ang R. P. Teo-Koh S. M. Kahlid A. (2004). Motivational predictors of young adolescents' participation in an outdoor adventure course: a self-determination theory approach. J. Adven. Educ. Outd. Learn. 4, 57–65. 10.1080/14729670485200421

[B70] Western B. Jackman S. (1994). Bayesian lnference for comparative research. Am. Polit. Sci. Rev. 88, 412–423. 10.2307/2944713

