# Supplementary material for: A Bayesian Mixed-Methods Analysis of Basic Psychological Needs Satisfaction through Outdoor Learning and Its Influence on Motivational Behavior in Science Class
Source: Front Psychol. 2017 Dec 19;8:2235. doi: 10.3389/fpsyg.2017.02235 (PMC5742242; doi:10.3389/fpsyg.2017.02235)
Supplement: Supplementary file 1 [file Presentation1.PDF]

Supplementary Material

A Bayesian Mixed-Methods Analysis of Basic Psychological Needs Satisfaction through Outdoor Learning and its Influence on Motivational Behavior in Science Class

Ulrich Dettweiler*, Gabriele Lauterbach, Christoph Becker, Perikles Simon

*** Correspondence:** Corresponding Author: ulrich.dettweiler@uis.no

1 Normal Distribution of Grades

Grades in Math and German

	Math	German
**Valid**	233	234
**Missing**	51	50
**Mean**	2.318	2.436
**Std. Deviation**	0.9480	0.8011
**Skewness**	0.4281	-0.1425
**Std. Error of Skewness**	0.1595	0.1591
**Kurtosis**	-0.2960	-0.5095
**Std. Error of Kurtosis**	0.3176	0.3169
**Minimum**	1	1
**Maximum**	5	4

In Germany, grades are from 1 (“very good”) to 6 (“insufficient”).

Supplementary Table 1 shows the descriptive values of grades in Math and German. The considerable low values for skewness and kurtosis hint at a fairly good normal distribution.

2 Model: SDI ~ A + C + RT + Gender

2.1 Bayes Factors for the Five Best Models

**Model**	**BF_{01}**	**Error %**
A + C + Gender	2.21	0.022
A + C + RT + Gender + Context	6.08	0.028
A + C + RS + RT + Gender	6.34	0.032
A + C + Gender + Context	12.63	0.034
A + C + RS + Gender	20.24	0.024
**Denominator: C + A + RT + Gender**		

Supplementary Table 2. The second to fifth best models compared to the best model as the denominator (SDI ~ A + C + RT + Gender). The Bayes Factors BF_{01} for the second to fifth models indicate the probability of not preferring those models over the first, which is by factor 2.21 more preferable than the second best, or 20.24 for the fifth best model. The error rate results from the sampling process of the posterior-distribution in the 10.000 MCMC chain-iterations, which can vary from analysis to analysis within the estimated error range.

2.2 Effect Analysis of Model Components

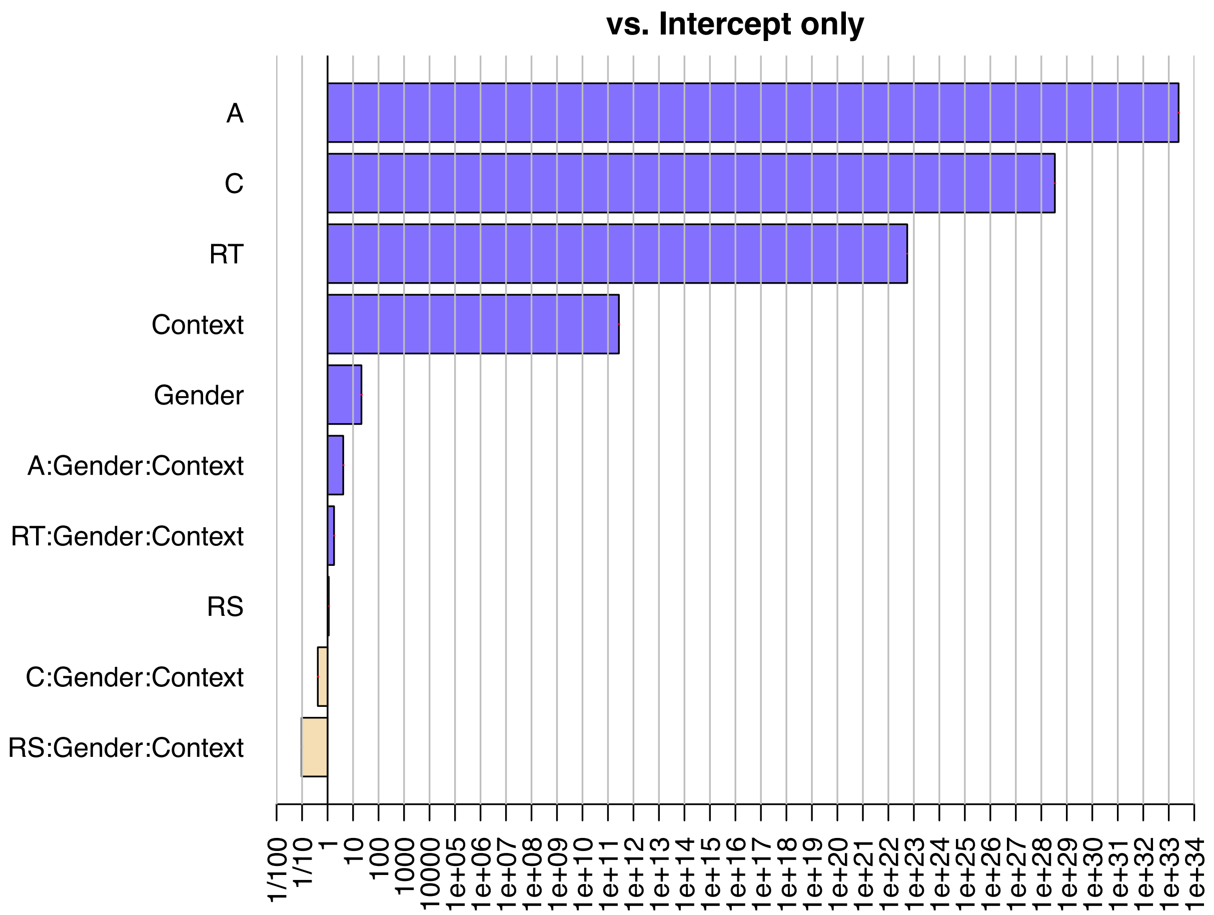

Supplementary Figure 1. Effect of the model components, indicating their relative importance. On the x-scale, the Bayes Factor BF_{10} is plotted for each component comparing the intercept-only model to the relevant models that can be formed by adding a covariate or the covariates interacting with the factors Gender and Context. In this component-by-component approach, Context does have an extremely high effect, since the outdoor-values are considerably higher than the indoor values – which is in line with the paired-samples comparisons.

2.3 MCMC-Diagnostics

	lower	upper
**mu**	5.422	5.935
**C-C**	0.512	1.646
**A-A**	0.674	1.817
**RT-RT**	0.126	0.980
**Gender-female**	0.246	0.761
**Gender-male**	-0.761	-0.246
**sig2**	8.026	10.226

Supplementary Table 3. Highest probability density intervals for the best model *SDI ~ A + C + RT + Gender*. For each parameter, the interval is constructed from the empirical cumulative distribution (eCDF) of the sample as the shortest interval for which the difference in the eCDF values of the endpoints is the nominal probability (Bates, 2006).

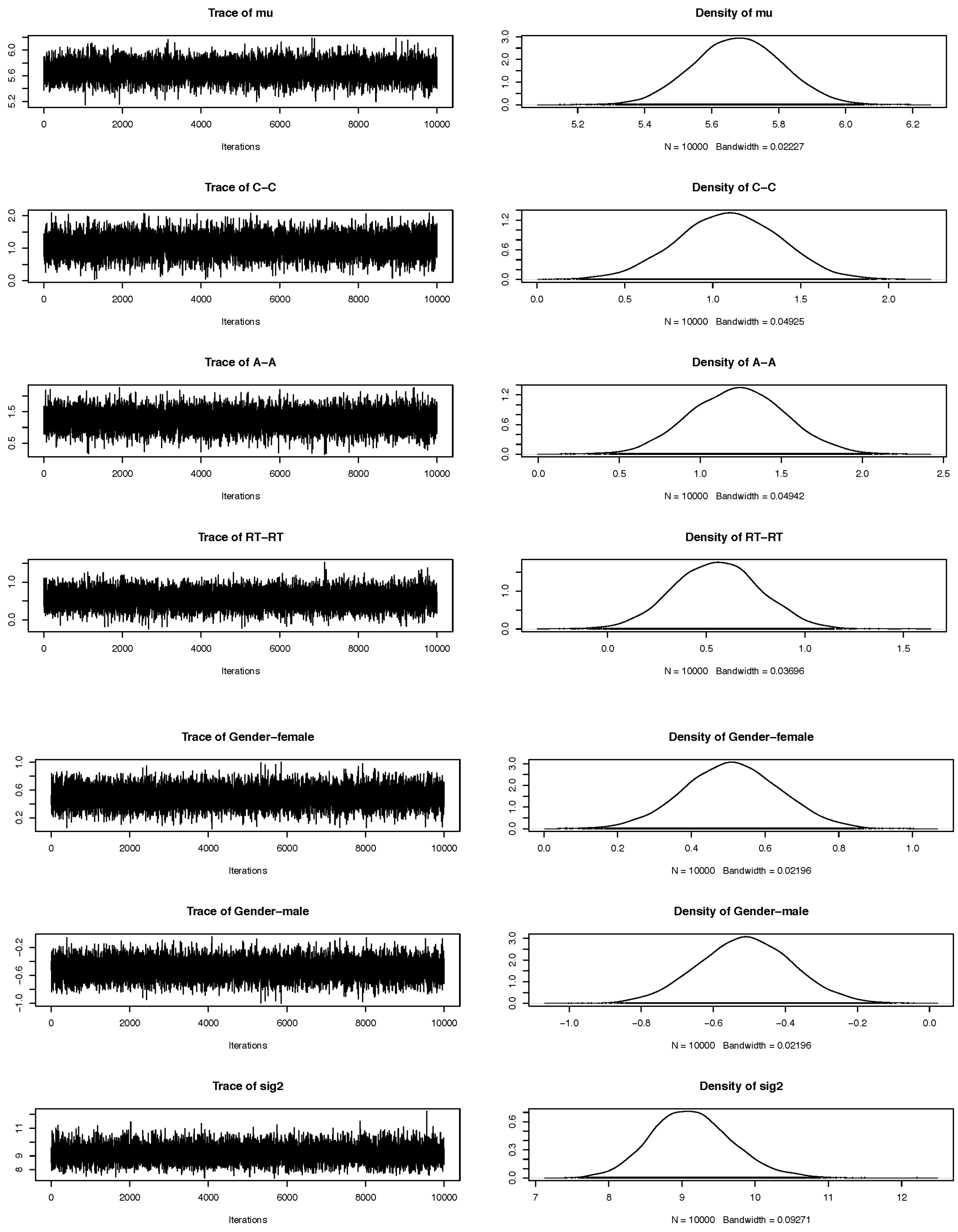

Supplementary Figure 2. 10.000 iteration chain plots and density intervals displayed for the model *SDI ~ A + C + RT + Gender*. The chains burn in regularly with a steady pattern indicating that the priori distribution is well calibrated and that the parameters are having sufficient state changes as the MCMC algorithm runs.

3 Model: SDI ~ diff C

3.1 Bayes Factors for the Five Best Models

**Model**	**BF_{01}**	**Error %**
diffC + diffA	2.81	< 0.001
diffC + diffRT	5.07	< 0.005
diffC + diffA + diffRT	5.35	< 0.001
diffC + Gender	6.40	0.031
diffC + diffRS	6.50	< 0.005
**Denominator: diffC**		

Supplementary Table 4. The second to fifth best models compared to the best model as the denominator (*diffSDI ~ diffC*). The Bayes Factors BF_{01} for the second to fifth models indicate the probability of not preferring those models over the first, which is by factor 2.81 more preferable than the second best, or 6.50 for the fifth best model. The error rate results from the sampling process of the posterior-distribution in the 10.000 MCMC chain-iterations, which can vary from analysis to analysis within the estimated error range.

3.2 Effect Analysis of Model Components

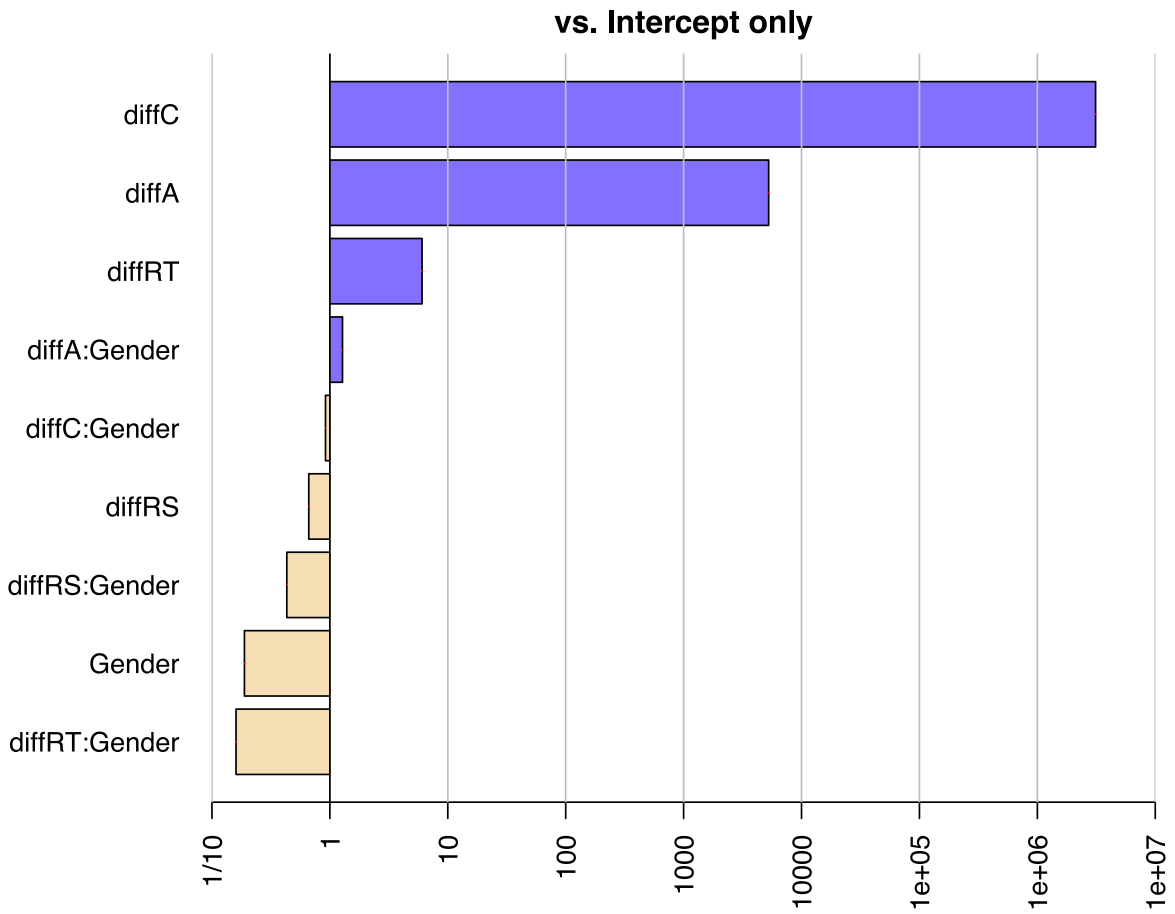

Supplementary Figure 3. Effect of the model components, indicating their relative importance. On the x-scale, the Bayes Factor BF_{10} is plotted for each component comparing the intercept-only model to the relevant models that can be formed by adding a covariate or the covariates interacting with the factor Gender. In this component-by-component approach, only diffC and diffA have strong effects. The evidence that no gender effect can be seen is moderate.

3.3 MCMC-Diagnostics

	lower	upper
diff_C	1.408	2.761
sig2	9.164	13.287

Supplementary Table 5. Highest probability density intervals for the best model *diffSDI ~ diff C*.

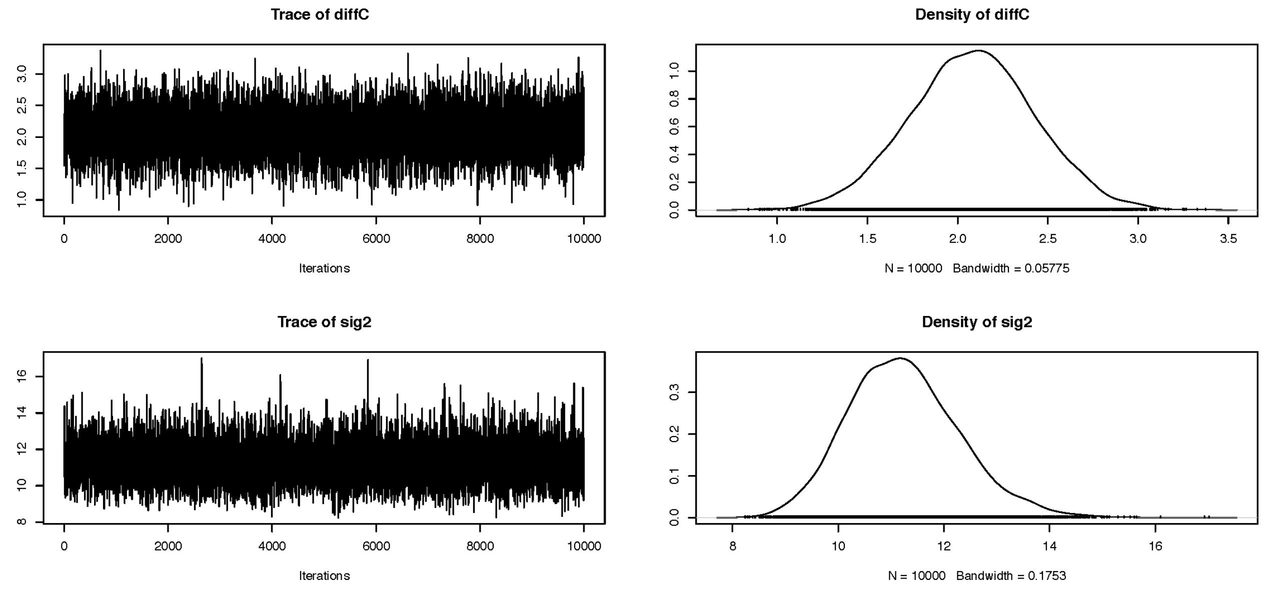

Supplementary Figure 4. 10.000 iteration chain plots and density intervals displayed for the model *diffSDI ~ diff C*. The chains burn in regularly with a steady pattern. The chains burn in regularly with a steady pattern indicating that the priori distribution is well calibrated and that the parameters are having sufficient state changes as the MCMC algorithm runs.

Reference:

Bates, D. (2006). CODA: Convergence Diagnosis and Output Analysis for MCMC. *R News* 6(1)**,** 7-11.
