# Supplementary material for: A Bayesian Mixed-Methods Analysis of Basic Psychological Needs Satisfaction through Outdoor Learning and Its Influence on Motivational Behavior in Science Class
Source: Front Psychol. 2017 Dec 19;8:2235. doi: 10.3389/fpsyg.2017.02235 (PMC5742242; doi:10.3389/fpsyg.2017.02235)
Supplement: Supplementary file 2 [file Image1.PDF]

The space-for-time approach as a didactical tool

Experiencing climate change during an Alpine educational research expedition

Ulrich Dettweiler[1,2], Gabriele Lauterbach[1,2], Barbara Mayer[1,2], Annette Menzel[3]

[1] School of Education, Research Group Outdoor Education & Experiential Learning, Technische Universität München
[2] Schülerforschungszentrum Berchtesgadener Land
[3] Wissenschaftszentrum Weihenstephan, Professorship Ecoclimatology, Technische Universität München

The hiking was trying, but it was great to observe how the trees and the plants changed [with the altitude]. (Tabea)

Building our own measurement tools was great for me, because we do not have the time to do such things at home. (Leonie)

Being together with the others was great! (Sabine)

I really liked that it was never boring, although we learned so much and did so many things. (Pablo)

I really liked the glacier, especially when we digged a hole there and took our own measurements under freezing conditions. (Lukas)

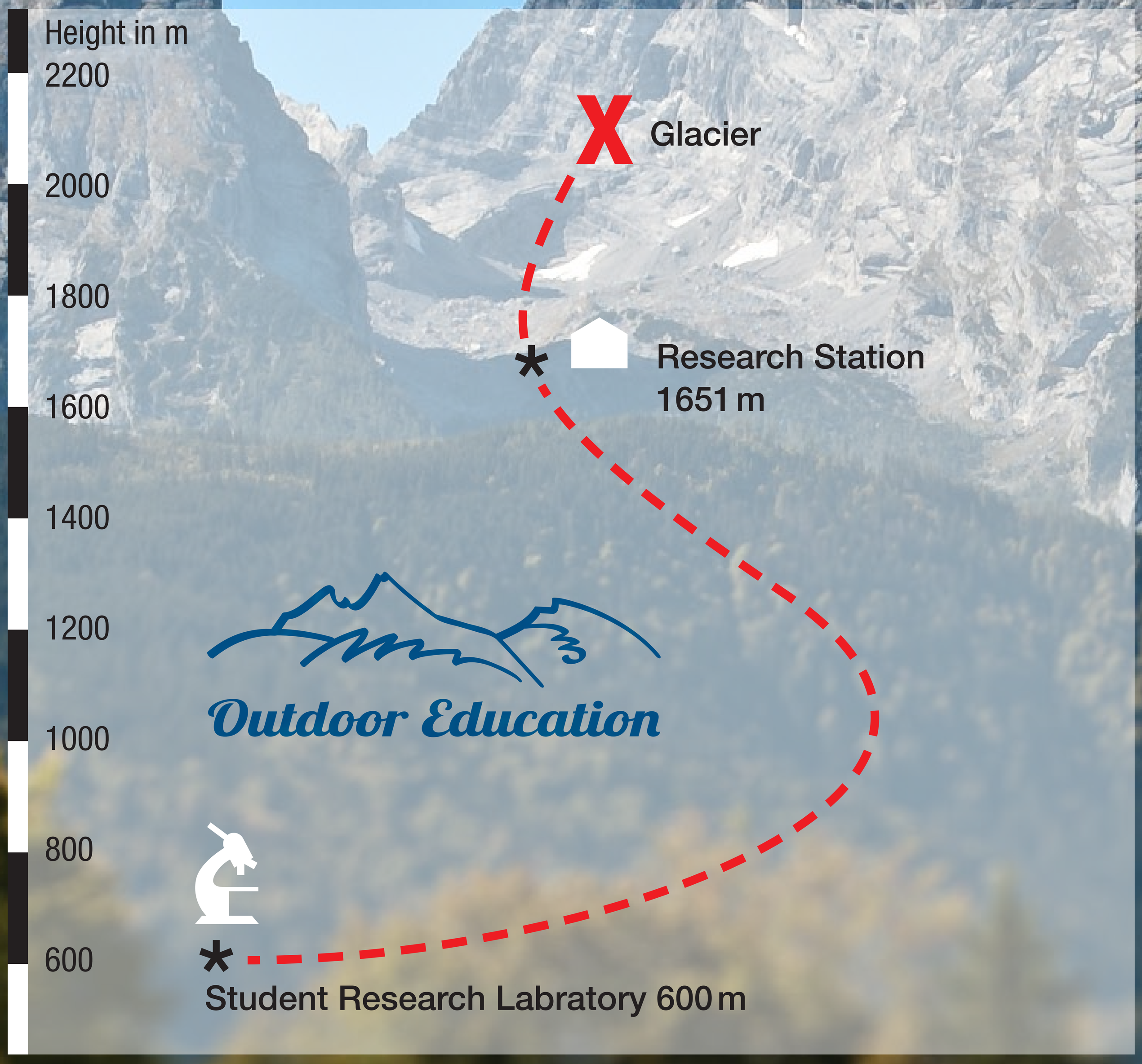

schülerFORSCHUNGSZENTRUM BERCHTESGADENER LAND

Expeditionary Learning Alpin (ELPIN$_{MINT}$)

Data-Collection in the Field / *Research Questions*

Botany (Plant biology)

Vegetation ecology – How do I identify a plant in the field and what does it tell me about its environment?
Pedology and abiotic factors – What are the influencing factors of a specific location for plant growth?
Plant phenology – How do plants change their timing with elevation?

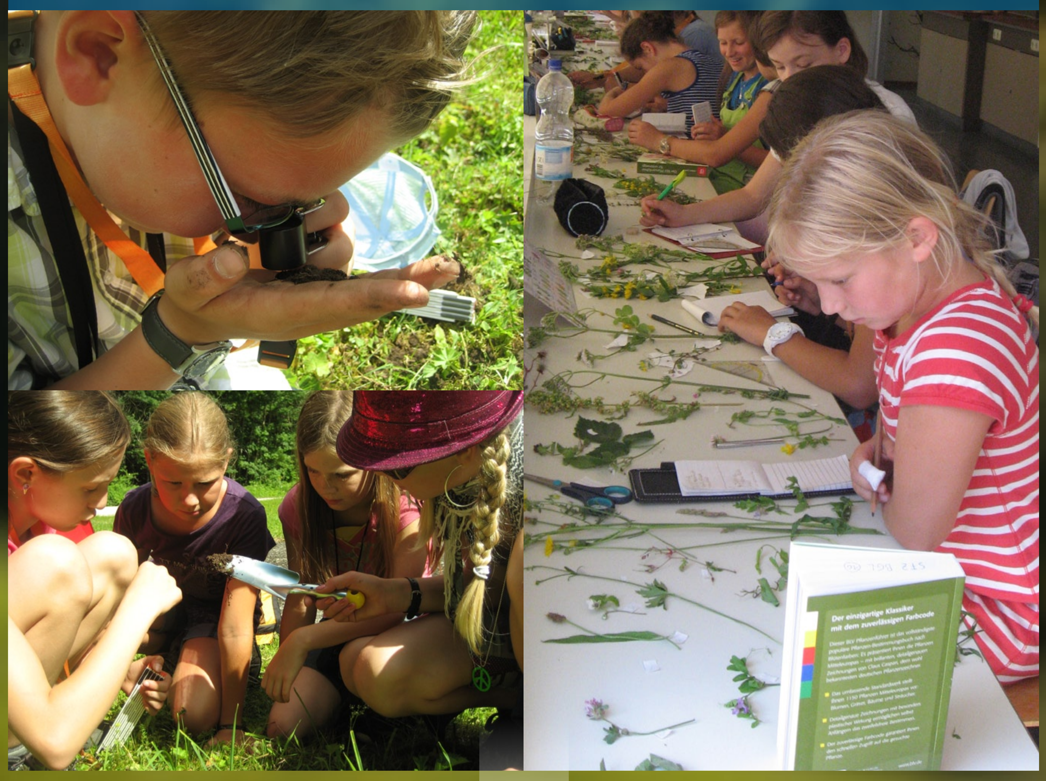

Social Learning

Group development – What are our group's values and principles?
Co-operation – What are positive factors of working as a team?
Personal growth – What personal insights do I take home with me?

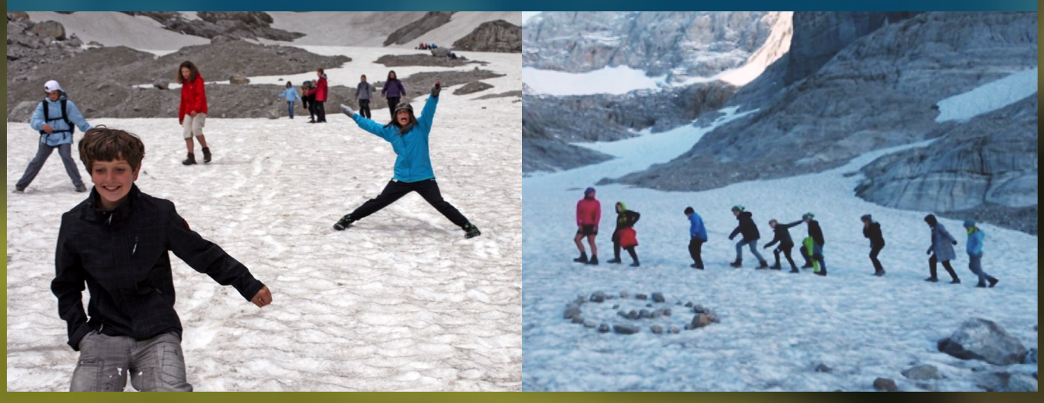

Meteorology and Landscape Ecology

Weather – What is weather and how can I measure it?
Climate – What is climate and how has it changed over the past decades?
Morphology – How do weather and water shape and change landscape and habitats?

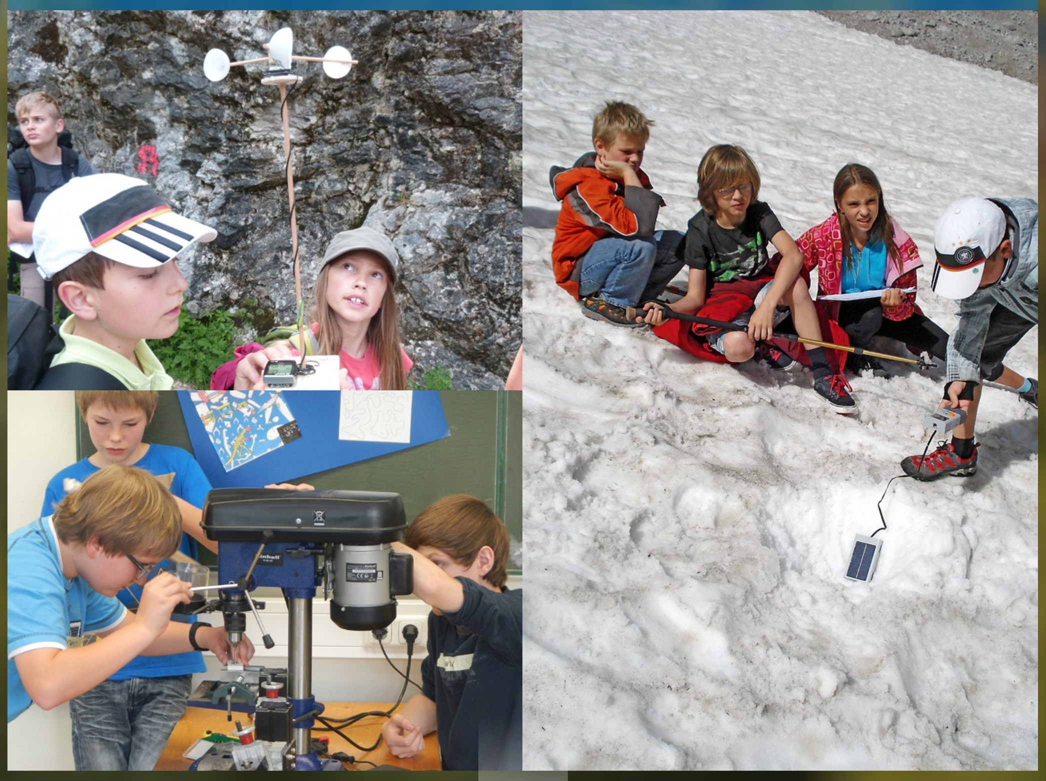

Data-Analysis in the Laboratory / *Research Methods*

Botany (Plant biology)

Individual Analyses in Sub-Groups

Displaying and understanding own vegetation surveys at three different altitudes

Plotting and explaining the distribution of different tree species against altitude

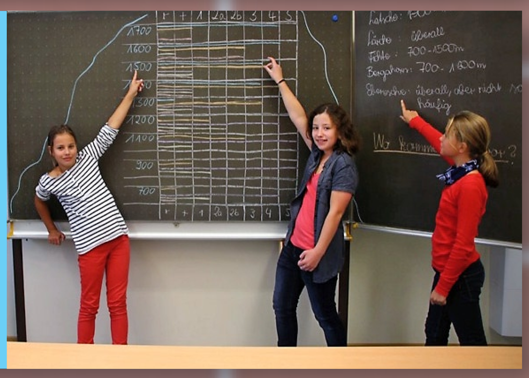

Plotting phenological onset dates of indicator plants against altitude and understanding the driving forces

Ecoclimatology

Merging the Sub-Groups

Mutual presentation of own methods in small merged groups / Learning by teaching

Presentation and discussion of results in group puzzle

Poster presentation

Transfer

Understanding recent climate change and its impact on the local habitats using the example of the "Blaueis"-glacier

Understanding future climate change by transferring the vertical spatial model into the temporal dimension

Meteorology and Landscape Ecology

Individual Analyses in Sub-Groups

Analyzing long-term climate data and understanding the recent data

Plotting and explaining actual weather data in dependency of altitude and exposition

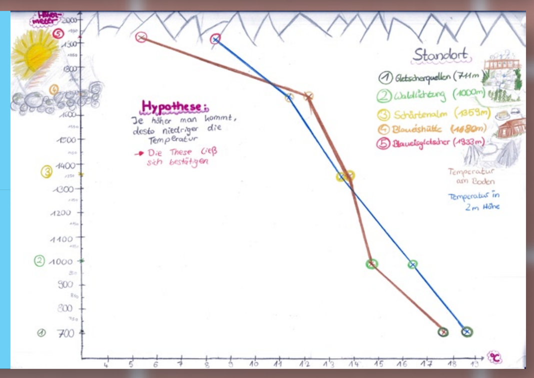

Evaluating data from self-made tools against electronic pro-tools

References

Dettweiler, U., Ünlü, A., Lauterbach, G., Becker, C., & Gschrey, B. (2015). Investigating the motivational behaviour of pupils during outdoor science teaching within self-determination theory. *Frontiers in Psychology, 6* (125). doi: 10.3389/fpsyg.2015.00125

Menzel, A., Sparks, T. H., Estrella, N., Koch, E., Aasa, A., Ahas, R., . . . Zust, A. N. A. (2006). European phenological response to climate change matches the warming pattern. *Global Change Biology, 12* (10), 1969-1976. doi: 10.1111/j.1365-2486.2006.01193.x

Schuster, C., Estrella, N., & Menzel, A. (2014). Shifting and extension of phenological periods with increasing temperature along elevational transects in southern Bavaria. *Plant Biology* 16, 332–344. doi: 10.1111/plb.12071

Schuster, C., Kirchner, M., Jakobi, G., & Menzel, A. (2013). Frequency of inversions affects senescence phenology of Acer pseudoplatanus and Fagus sylvatica. *International Journal of Biometeorology* (58), 485–498. doi: 10.1007/s00484-013-0709-0

GOTHENBURG JUN 29 - JUL 2 2015
8TH WEEC Planet and People – how can they develop together?

Robert Bosch Stiftung

School of Education

Arbeitsgruppe Outdoor Education
Arcisstraße 21
80333 München
www.outdoor.edu.tum.de

Technische Universität München
